# Diagnostic accuracy of CT for identifying high-risk colon cancer: a systematic review and meta-analysis

**DOI:** 10.1007/s00330-025-11844-2

**Published:** 2025-08-16

**Authors:** Jona Shkurti, Kim van den Berg, Renaud L. M. Tissier, Stevie van der Mierden, Max J. Lahaye, Regina G. H. Beets-Tan, Joost Nederend

**Affiliations:** 1 Department of Diagnostic Oncology, The Netherlands Cancer Institute–Antoni van Leeuwenhoek Hospital, Amsterdam, The Netherlands; 2 GROW School for Oncology and Reproduction, Maastricht University, Maastricht, The Netherlands; 3 Catharina Cancer Institute, Catharina Hospital, Eindhoven, The Netherlands; 4 Biostatistics Center, The Netherlands Cancer Institute–Antoni van Leeuwenhoek Hospital, Amsterdam, The Netherlands; 5 Scientific Information Service, The Netherlands Cancer Institute–Antoni van Leeuwenhoek Hospital, Amsterdam, The Netherlands; 6 Institute of Regional Health Research, University of Southern Denmark, Odense, Denmark; 7 Department of Radiology, Catharina Hospital, Eindhoven, The Netherlands

**Keywords:** Colonic neoplasms, X-ray computed tomography, Neoplasm staging, Neoadjuvant therapy

## Abstract

**Objectives:**

This systematic review and meta-analysis aimed to assess the diagnostic accuracy of CT in differentiating high-risk from low-risk colon cancer, with a focus on staging parameters and the impact of CT slice thickness.

**Materials and methods:**

A systematic search of Ovid MEDLINE and Embase.com was conducted from January 1, 2015, to September 24, 2024, to identify studies evaluating CT-based staging accuracy using histopathology as the reference standard. The QUADAS-2 tool assessed the risk of bias. Pooled sensitivity, specificity, and diagnostic odds ratio (DOR) were calculated using a bivariate random-effects model. Subgroup analyses explored the influence of CT techniques, slice thickness, and study design on diagnostic performance.

**Results:**

The meta-analysis included forty-four studies. CT demonstrated 83% sensitivity (95% CI, 79–86%) and 70% specificity (95% CI, 66–74%) for detecting pT3-T4 tumors (DOR: 10.0). For pT3cd-T4 (> 5 mm muscularis propria invasion), sensitivity was 67% (61–73%), specificity 88% (83–92%) and DOR 13.7 (9.0–21.0). Detection of pN+ yielded 64% sensitivity (60–68%), 67% specificity (62–72%) and DOR of 3.5 (3.0–4.2). Sensitivity for extramural venous invasion (EMVI+) was 49% (41–56%), with 77% specificity (67–84%) and DOR 3.0 (2.0–4.4). Studies with < 5 mm slice thickness showed higher sensitivity but lower specificity. High I² values indicated substantial heterogeneity across studies.

**Conclusion:**

CT demonstrates high sensitivity for detecting T3-T4 colon cancer but moderate sensitivity for nodal involvement and EMVI+. Diagnostic performance varies with technical factors, emphasizing the need for standardized imaging protocols and supplementary diagnostic tools to improve colon cancer staging.

**Registration:**

PROSPERO (International Prospective Register of Systematic Reviews) CRD42022374615.

**Key Points:**

***Question*** *Accurate CT staging is crucial for guiding neoadjuvant therapy in colon cancer, but its ability to distinguish high-risk from low-risk cases remains uncertain.*

***Findings*** *CT showed high sensitivity for distinguishing pT3-T4 tumors but only moderate sensitivity for pT3cd-T4, nodal involvement, and extramural venous invasion.*

***Clinical relevance*** *This systematic review critically evaluates CT diagnostic accuracy in colon cancer staging, revealing its strengths and limitations. The findings highlight the need for optimized imaging protocols and complementary tools to enhance risk stratification and guide clinical decisions.*

## Introduction

Surgical resection followed by adjuvant chemotherapy remains the standard treatment for most patients with colon cancer [1], with histopathological evaluation playing a central role in guiding therapy. In recent years, neoadjuvant treatment approaches have been explored for selected patients with locally advanced colon cancer [2, 3]. Several clinical trials investigating neoadjuvant chemotherapy or immunotherapy in patients with T3-4 or N+ tumors have shown promising results, with improvements in tumor downstaging and resectability [3–9]. However, a shift from the conventional adjuvant chemotherapy strategy to neoadjuvant treatment would require radiological risk stratification, rather than histopathological evaluation, to guide treatment decisions.

In contrast to rectal cancer, where the role of MRI to select high-risk tumors for neoadjuvant treatment is established and part of the guidelines, colon cancer continues to rely on CT for preoperative staging. The use of MRI in colon cancer is limited to research settings, due to anatomical, technical, economical and accessibility limitations [10]. However, CT accuracy in detecting high-risk colon tumors has been a subject of ongoing debate and research [11, 12].

Previous studies have reported varying degrees of sensitivity and specificity of CT in the detection of high-risk features such as tumor invasion beyond the bowel wall (pT3-4), deep invasion beyond 5 mm of muscularis propria (pT3cd-T4), nodal involvement (pN+), extramural venous invasion (EMVI), and tumor deposits. A meta-analysis by Nerad et al described a pooled sensitivity of 90% for detecting pT3-T4 tumors [11]. However, the sensitivity was 77% for detecting pT3cd-T4 tumors and 71% for detecting nodal involvement. These results were based on studies published before 2015. Since then, colon cancer staging has gained further attention, particularly with the growing interest in neoadjuvant treatment regimens [3, 9].

Analyzing data from more recent studies allows for the evaluation of the strengths and limitations of CT in colon cancer staging and may help identify opportunities for enhancing imaging protocols and technologies. This systematic review and meta-analysis aimed to assess the diagnostic performance of CT in distinguishing high-risk from low-risk colon cancer, particularly focusing on its ability to detect high-risk features such as pT3-T4, pT3cd-T4, nodal involvement, EMVI, and tumor deposits. By addressing these aspects, this study seeks to clarify the role of CT in the local staging of colon cancer and support clinicians in optimizing treatment strategies.

## Methods

This systematic review was conducted and reported in accordance with the Preferred Reporting Items for Systematic reviews and Meta-Analyses–Diagnostic Test Accuracy (PRISMA–DTA) statement [13]. A review protocol was registered in PROSPERO (International Prospective Register of Systematic Reviews; CRD42022374615) in advance [14].

### Literature search and eligibility criteria

The electronic bibliographic databases, Ovid MEDLINE and Embase.com, were systematically searched to identify relevant studies. The search strategy was schematically as follows: “colon cancer” AND “cancer staging” AND (“CT” OR “EMVI” OR “tumor deposits”). Complete search details are provided in Appendix S1. Since the 2016 review by Nerad et al [11] had already evaluated TN staging using CT, the term “CT” was searched only for studies published from 1 January 2015 onward. For the other two subjects, EMVI and tumor deposits, searches were conducted from the inception of the database. No restrictions were applied regarding patient age or gender. Duplicate records were removed by deduplication in EndNote, followed by a manual review in Rayyan.

Articles written in languages other than English, Dutch, Italian, Spanish, French, and German were translated using Google Translate (Google LLC). Articles that could not be successfully translated were excluded. The original literature searches were run on 25 May 2022 and subsequently updated on 11 November 2022, 11 July 2023, 26 July 2024, and 24 September 2024. To ensure comprehensive coverage, the citations of the most relevant studies were also screened for additional eligible studies.

Original studies assessing the diagnostic accuracy of CT for primary staging of colon cancer with histopathological examination serving as the reference standard were included. Exclusion criteria comprised reviews, meta-analyses, conference abstracts, posters, case reports, expert opinions, and letters to the editor. Studies involving patients without primary colon cancer or those assessing imaging modalities other than CT were also excluded.

### Main outcomes

The primary outcome was the accuracy of CT in detecting tumor invasion beyond the bowel wall, locoregional nodal involvement, EMVI, and tumor deposits in colon cancer, using histopathological evaluation as the reference standard. The secondary outcome was the difference in the diagnostic performance of CT based on slice thickness, CT technique or study type (prospective or retrospective). Measures of effect included hierarchical summary receiver operating characteristic (HSROC) curve, pooled sensitivity, pooled specificity, and diagnostic odds ratio (DOR).

### Data extraction

Articles were extracted from the aforementioned databases based on the literature search. Two reviewers (J.S., K.B.) independently screened all articles based on the title and abstract to determine their eligibility. The remaining articles were evaluated based on the full text. In case of disagreements between the reviewers, a discussion took place to reach consensus. In case of persistent disagreements, an independent reviewer (J.N.) was decisive in the exclusion or selection of the articles. The study selection process is presented in the PRISMA flowchart [15].

The included articles were evaluated for relevant data. Data on patient demographics, technical CT features, diagnostic performance, and type of study (prospective vs. retrospective) were extracted. If a study did not state true positive (TP), false positive (FP), true negative (TN), or false negative (FN) results, they were derived from marginal totals or from sensitivity and specificity. When these results could not be derived from the study, the corresponding authors of the respective studies were contacted to provide these results. In the case of no response, a reminder was sent 2 weeks after the initial attempt.

### Risk of bias (quality) assessment

The methodological quality of the eligible articles was assessed individually using the revised tool for the Quality Assessment of Diagnostic Accuracy Studies (QUADAS) [16] by the two previously mentioned investigators (J.S., K.B.) independently, resolving disagreements through discussion. If consensus was not reached, an independent reviewer (J.N.) was the decisive party.

### Strategy for data synthesis

Two-by-two contingency tables were constructed for tumor invasion through the bowel wall (T1–T2 vs. T3–T4), tumor invasion depth less than 5 mm vs. 5 mm or greater in the pericolic fat (T1–T3ab vs. T3cd–T4), locoregional nodal involvement (N0 vs. N+), and extramural venous invasion (EMVI+ vs. EMVI−). The outcomes were all calculated on a per-lesion basis. For studies that reported results for multiple observers, each observation was treated as a separate study [17].

Estimates of sensitivity, specificity, and DOR were derived by means of the bivariate random-effects model from individual studies or observers and are presented in plots. A hierarchical summary ROC model was used to construct the HSROC curve and calculate summary estimates of sensitivity, specificity, and DOR [18], as the meta-analysis accounted for heterogeneity between studies via the use of random-effects models. The heterogeneity of the study results was evaluated by calculating the Higgins I² statistic, with I^2^ of ≤ 50%, 50% < I^2^ ≤ 75%, and > 75% classified as low, medium, and high heterogeneity, respectively [19, 20].

Publication bias was explored using the Deeks funnel plot asymmetry test [21]. A *p* < 0.10 for the slope coefficient was considered to indicate statistically significant asymmetry. If there was proof of publication bias, selection model methods (such as p-curve or p-uniform) were used to correct it.

Subgroup analyses were performed for studies with a CT slice thickness < 5 mm versus 5–10 mm, to evaluate whether slice thickness affects the diagnostic performance of CT. Subgroup analyses were also performed for retrospective vs. prospective studies, as well as for CT techniques.

The meta-analysis was performed with the packages meta (v 7.0) and mada (v 0.5.11) from the R software (v 4.3.0).

## Results

### Literature search

**Fig. 1:**
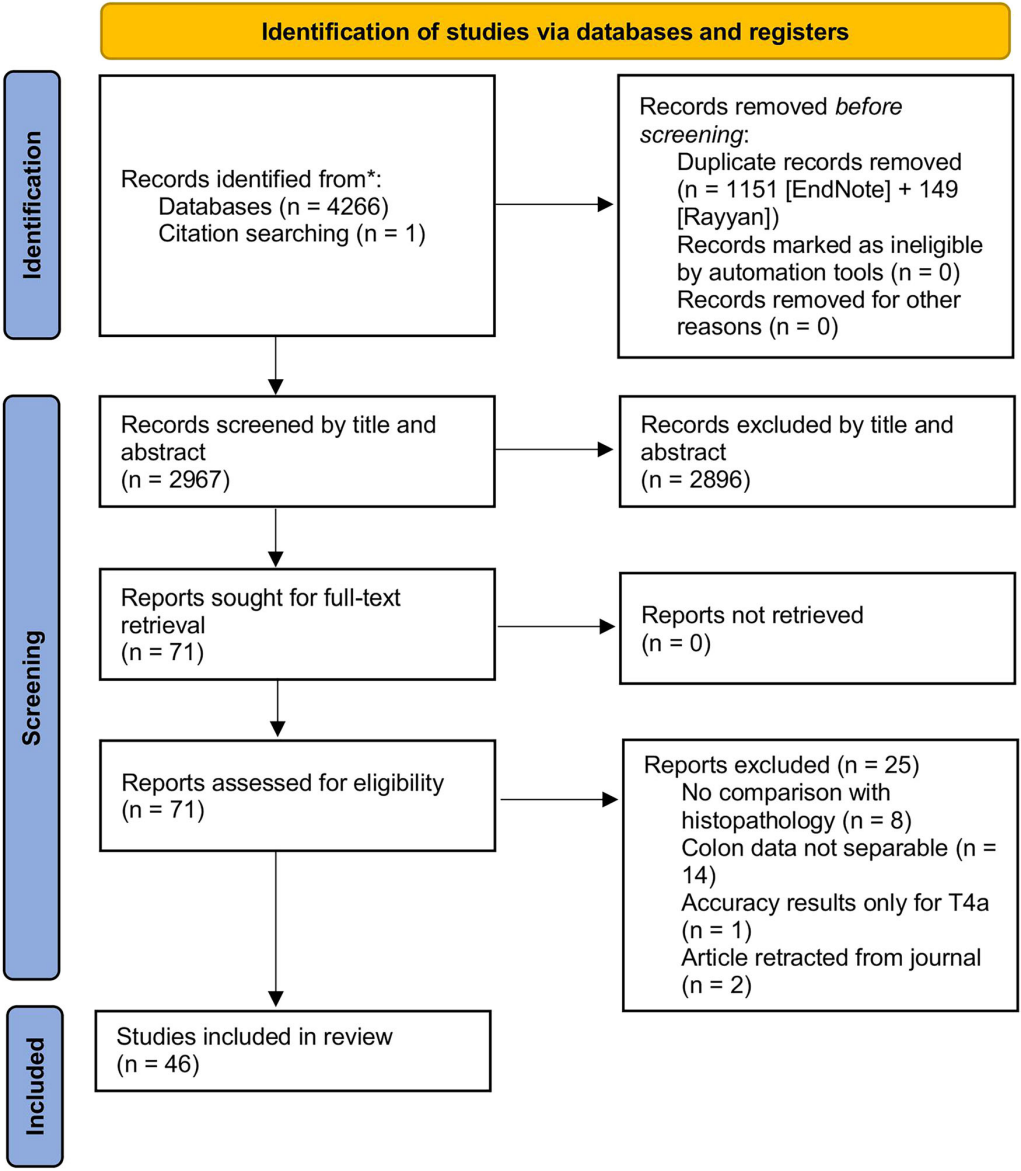
PRISMA flowchart depicting the inclusion of patients

The initial search yielded 4266 articles, and 2967 articles remained after the removal of duplicates (Fig. 1). These articles were screened by title and abstract. After screening, 74 articles were selected for full-text review. Another 27 studies were excluded because they did not meet the inclusion criteria. Of the 46 studies [12, 22–66] included in the systematic review, 2 studies [23, 29] were excluded from the meta-analysis due to incomplete diagnostic data (missing TP, TN, FP, FN values), even after contacting the authors of the studies. These studies were still considered in the qualitative synthesis to provide a comprehensive overview. In total, 44 studies remained for inclusion in the meta-analysis. The characteristics of all studies included in the systematic review are shown in Tables S1 and S2. For the T-category analysis of T1–T2 vs. T3–T4, 30 articles [25–27, 30, 31, 33–45, 47–51, 54, 56, 58, 59, 61, 65, 66] with 51 observations qualified (86,053 patients). Nine articles [25, 36, 40, 41, 55, 59, 60, 63, 66] with 15 observations qualified for the T-category analysis of T1–T3ab vs. T3cd–T4 (5068 patients). In the nodal involvement analysis, 35 articles [22, 24–28, 30–39, 42, 44–49, 52, 54, 55, 57, 59–66] with 61 observations were included (96,741 patients). Nine articles [12, 36, 37, 39, 52, 53, 61, 63, 66] with 15 observations qualified for the EMVI+ vs. EMVI− category (1500 patients). Thirty articles were used in more than one category, explaining the overlapping references between the categories.

### Quality assessment

To assess potential publication bias, a linear regression test of funnel plot asymmetry was conducted. The test result indicated no significant evidence of asymmetry (t = −0.82, df = 59, *p*-value = 0.42), suggesting no substantial publication bias. The estimated bias is −0.2975 (SE = 0.3628). The multiplicative residual heterogeneity variance (tau²) was 6.0210, indicating a high level of between-study variability. The analysis was performed using the standard error of the effect size as a predictor and by applying an inverse-variance weighting method, as recommended by Harbord et al [67].

**Fig. 2:**
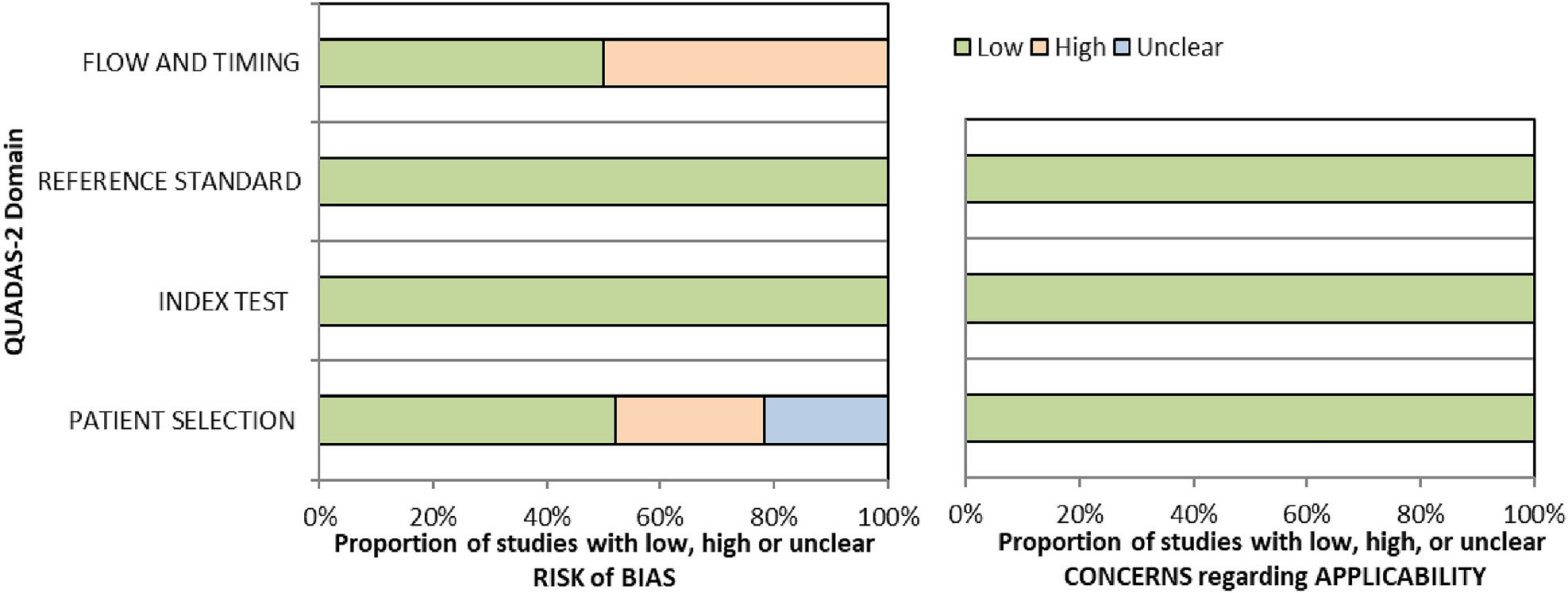
QUADAS-2 results: an overview of 46 studies that fulfilled the inclusion criteria

The results of the assessment using the QUADAS-2 checklist are shown in Fig. 2. The full QUADAS-2 assessment on a per-study basis is shown in Fig. S1.

### Differentiating T1–T2 from T3–T4 tumors

**Fig. 3:**
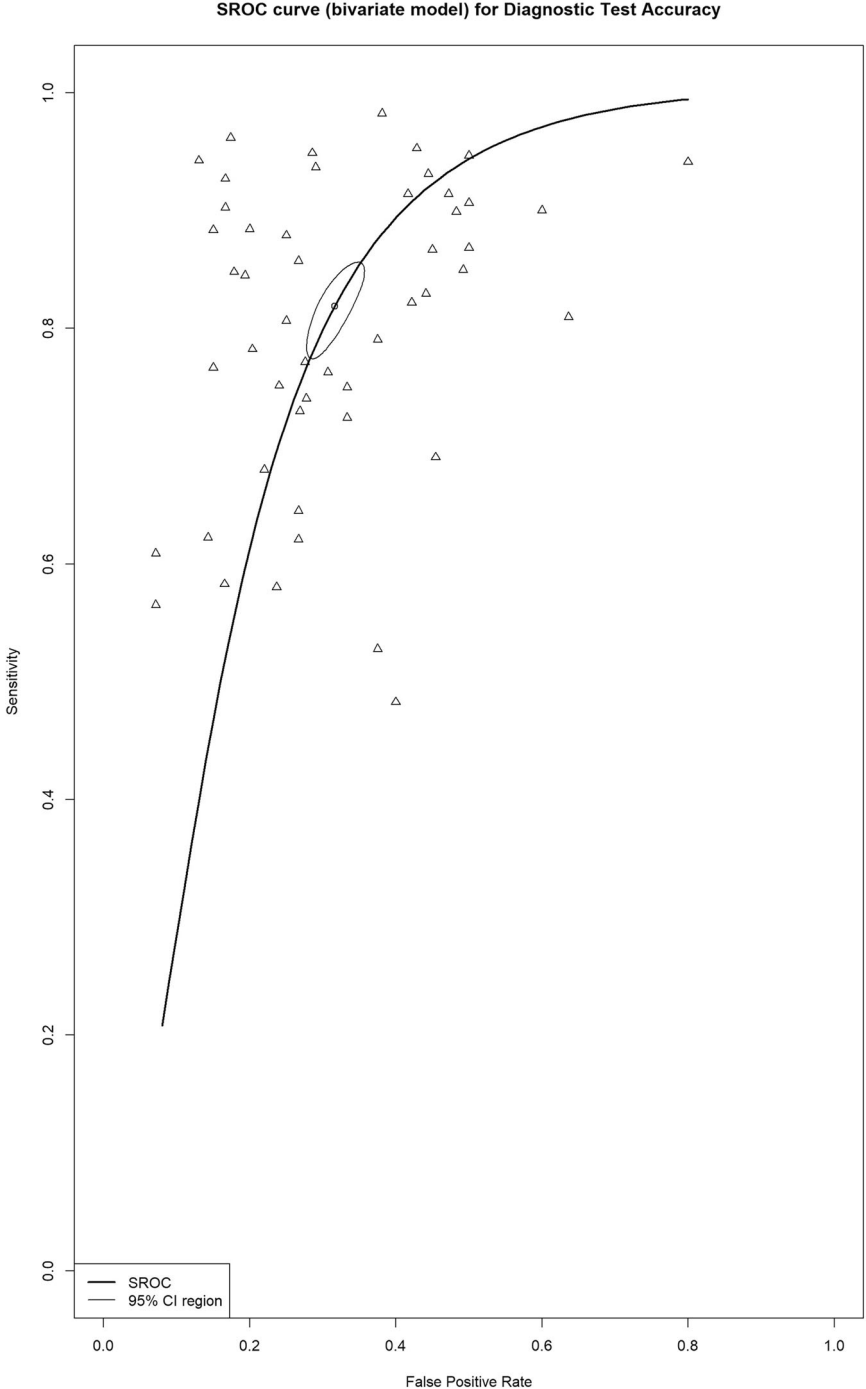
Bivariate summary estimates of sensitivity and specificity for all studies that evaluated the accuracy of detecting tumor invasion for T1–T2 vs. T3–T4. Sensitivity and specificity were plotted in receiver operating characteristic (ROC) space for individual studies. Hierarchical summary receiver operating characteristic (HSROC) curve was plotted through data points

The summary estimates for sensitivity and specificity for the detection of tumor growth beyond the bowel wall (T3–T4 tumors) were 83% (95% CI, 79–86%) and 70% (95% CI, 66–74%), respectively (Fig. 3), with a DOR of 10.0 (95% CI, 8.2–12.1). The I^2^ value was 91% (95% CI, 89–93%) for sensitivity, 74% (95% CI, 66–81%) for specificity, and 61% (95% CI, 46–71%) for DOR. Forest plots of sensitivity and specificity, along with their respective heterogeneity, are presented in Fig. S2.

In the study by Wang et al [51], two different criteria for local staging were used to measure accuracy for this category: the conventional criteria (8th pathologic TNM) and new criteria (based on membrane anatomy and visceral adipose tissue). A study by Platt et al reported separate results for proficient mismatch repair (pMMR) and deficient MMR (dMMR) tumors [49]. Venara et al reported the results of two readers: a senior abdominal radiologist with more than 10 years of experience in reading water-enema multidetector CT (WE-MDCT), and a surgeon with no experience in reading WE-MDCT [45].

In studies using a slice thickness of < 5 mm [25, 27, 30, 31, 33, 34, 38–40, 44, 47, 49, 51, 56, 58, 59, 61, 65, 66], the summary estimates for sensitivity and specificity for detecting tumor growth beyond the bowel wall were 84% (95% CI, 80–87%) and 68% (95% CI, 63–73%), respectively, with a DOR of 10.5 (95% CI, 8.1–13.5). The I^2^ value was 88% (95% CI, 84–90%) for sensitivity and 62% (95% CI, 46–73%) for specificity.

There were no available studies using a slice thickness > 5 mm for this category, and one study provided no information on slice thickness. The results of the other subgroup analyses are shown in Table 1.

**Table 1:** Subgroup analysis for all comparison categories based on CT technique and study design

Category, characteristic	Sensitivity, % (95% CI)	Specificity, % (95% CI)	No. of lesions	No. of observations	No. of studies
T1–T2 vs. T3–T4	83% (79–86%)	70% (66–74%)	86,063	51	30
CT technique					
Helical	-	-	-	-	-
MDCT	84% (80–87%)	70% (66–74%)	75,363	47	26
NA	73% (60–83%)	74% (66–80%)	10,700	4	4
Study design					
Retrospective	84% (79–87%)	69% (64–73%)	84,563	31	22
Prospective	82% (74–88%)	74% (65–81%)	1424	18	7
T1-3ab vs. T3cd-4	67% (61–73%)	88% (83–92%)	5068	15	9
CT technique					
Helical	-	-	-	-	-
MDCT	67% (61–73%)	88% (83–92%)	5068	15	9
NA	-	-	-	-	-
Study design					
Retrospective	69% (62–76%)	92% (85–96%)	4495	7	5
Prospective	57% (39–73%)	84% (76–90%)	497	6	3
N0 vs. N+	64% (60–68%)	67% (62–72%)	96,761	61	35
CT technique					
Helical	-	-	-	-	-
MDCT	65% (60–69%)	67% (60–72%)	82,203	52	29
NA	58% (51–65%)	72% (66–78%)	14,558	9	6
Study design					
Retrospective	64% (59–69%)	68% (60–75%)	95,066	40	26
Prospective	61% (56–65%)	67% (60–73%)	1619	19	8
EMVI− vs. EMVI+	49% (41–56%)	77% (67–84%)	1498	15	9
CT technique					
Helical*	-	-	54	2	1
MDCT	48% (40–57%)	75% (64–84%)	1294	12	7
NA	-	-	150	1	1
Study design					
Retrospective	48% (39–58%)	79% (72–85%)	930	8	6
Prospective	49% (35–62%)	72% (46–89%)	492	5	2

*NA* not available

* There were not enough observations to perform a meta-analysis

### Differentiating T1–T3ab from T3cd–T4 tumors

**Fig. 4:**
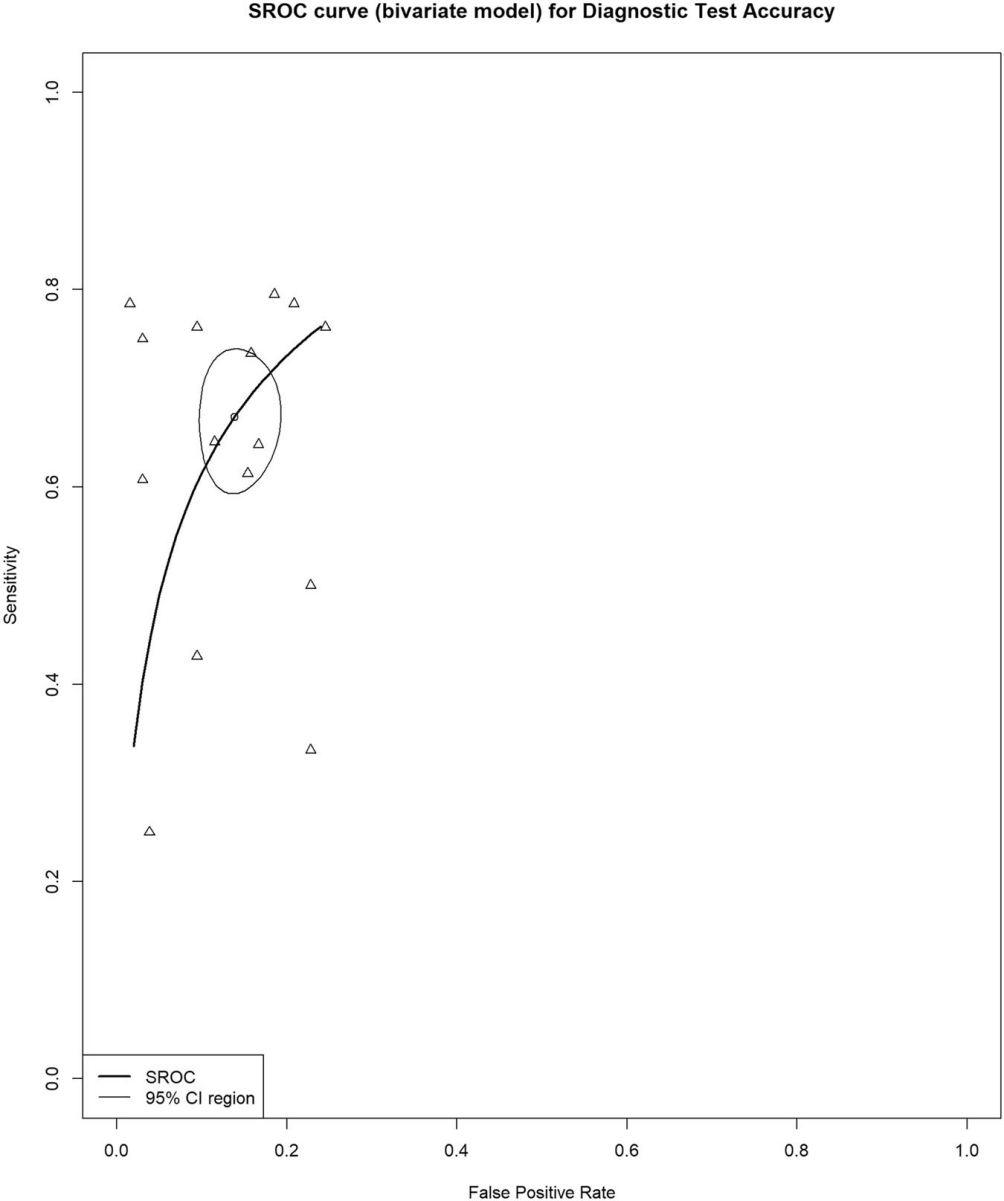
Bivariate summary estimates of sensitivity and specificity for all studies that evaluated the accuracy of detecting tumor invasion for T1–T3ab versus T3cd–T4. Sensitivity and specificity were plotted in receiver operating characteristic (ROC) space for individual studies. Hierarchical summary receiver operating characteristic (HSROC) curve was plotted through data points

The summary estimates for sensitivity and specificity for the detection of tumor invasion to a depth of 5 mm or more beyond the bowel wall were 67% (95% CI, 61–73%) and 88% (95% CI, 83–92%), respectively, with a DOR of 13.7 (95% CI, 9.0–21.0) (Fig. 4). The I^2^ value was 52% (95% CI, 13–73%) for sensitivity, 66% (95% CI, 41–80%) for specificity, and 59% (95% CI, 28–77%) for DOR. Two studies [30, 33] were excluded from the meta-analysis for this category due to the unavailability of TP, TN, FP, and FN values. Forest plots of sensitivity and specificity, along with their respective heterogeneity, are presented in Fig. S3.

In studies using a slice thickness of < 5 mm [25, 36, 40, 55, 59, 63, 66], the summary estimates for sensitivity and specificity for detecting tumor growth beyond the bowel wall were 69% (95% CI, 62–75%) and 88% (95% CI, 82–92%), respectively, with a DOR of 14.3 (95% CI, 8.3–24.8). The I^2^ value was 55% (95% CI, 17–76%) for sensitivity and 65% (95% CI, 36–81%) for specificity.

There were no available studies using a slice thickness of 5 mm or greater for this category.

### Nodal involvement

**Fig. 5:**
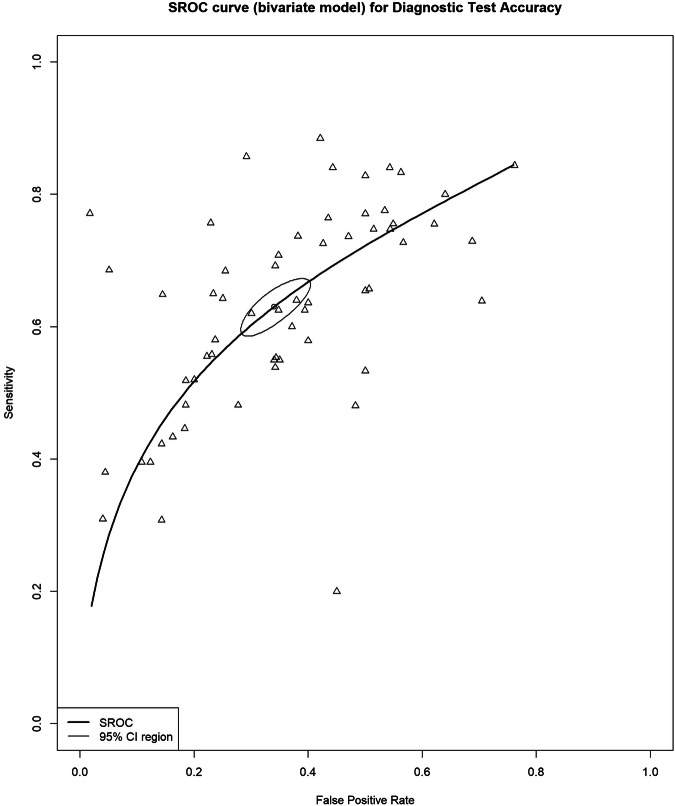
Bivariate summary estimates of sensitivity and specificity for all studies that evaluated the accuracy of detecting nodal involvement. Sensitivity and specificity were plotted in receiver operating characteristic (ROC) space for individual studies. Hierarchical summary receiver operating characteristic (HSROC) curve was plotted through data points

The summary estimates for sensitivity and specificity of nodal involvement (N0 vs. N+) were 64% (95% CI, 60–68%) and 67% (95% CI, 62–72%), respectively. The DOR for detecting nodal involvement was 3.6 (95% CI, 3.0–4.2) (Fig. 5). The I^2^ value was 96% (95% CI, 95–96%) for sensitivity and 97% (95% CI, 97–98%) for specificity. Forest plots of sensitivity and specificity, along with their respective heterogeneity, are presented in Fig. S4.

The results of Leonhardi et al were based on three different criteria for lymph node metastases: > 10 mm short axis diameters, NODE-RADS score > 3, and NODE-RADS score > 4 [64]. Choi et al reported results of three observers: the original radiologist, a second radiologist, and a surgeon [24].

In studies using a slice thickness of < 5 mm [25, 27, 28, 30–32, 34, 36, 38, 39, 44, 47, 49, 52, 55, 59, 61, 63–66], the summary estimates for sensitivity and specificity for detecting nodal involvement were 67% (95% CI, 62–71%) and 66% (95% CI, 58–72%), respectively, with a DOR of 3.7 (95% CI, 2.9–4.6). The I^2^ value was 94% (95% CI, 92–95%) for sensitivity and 96% (95% CI, 95–97%) for specificity.

Only two studies used a slice thickness of ≥ 5 mm in this category [33, 46].

### Differentiating EMVI+ from EMVI− tumors

**Fig. 6:**
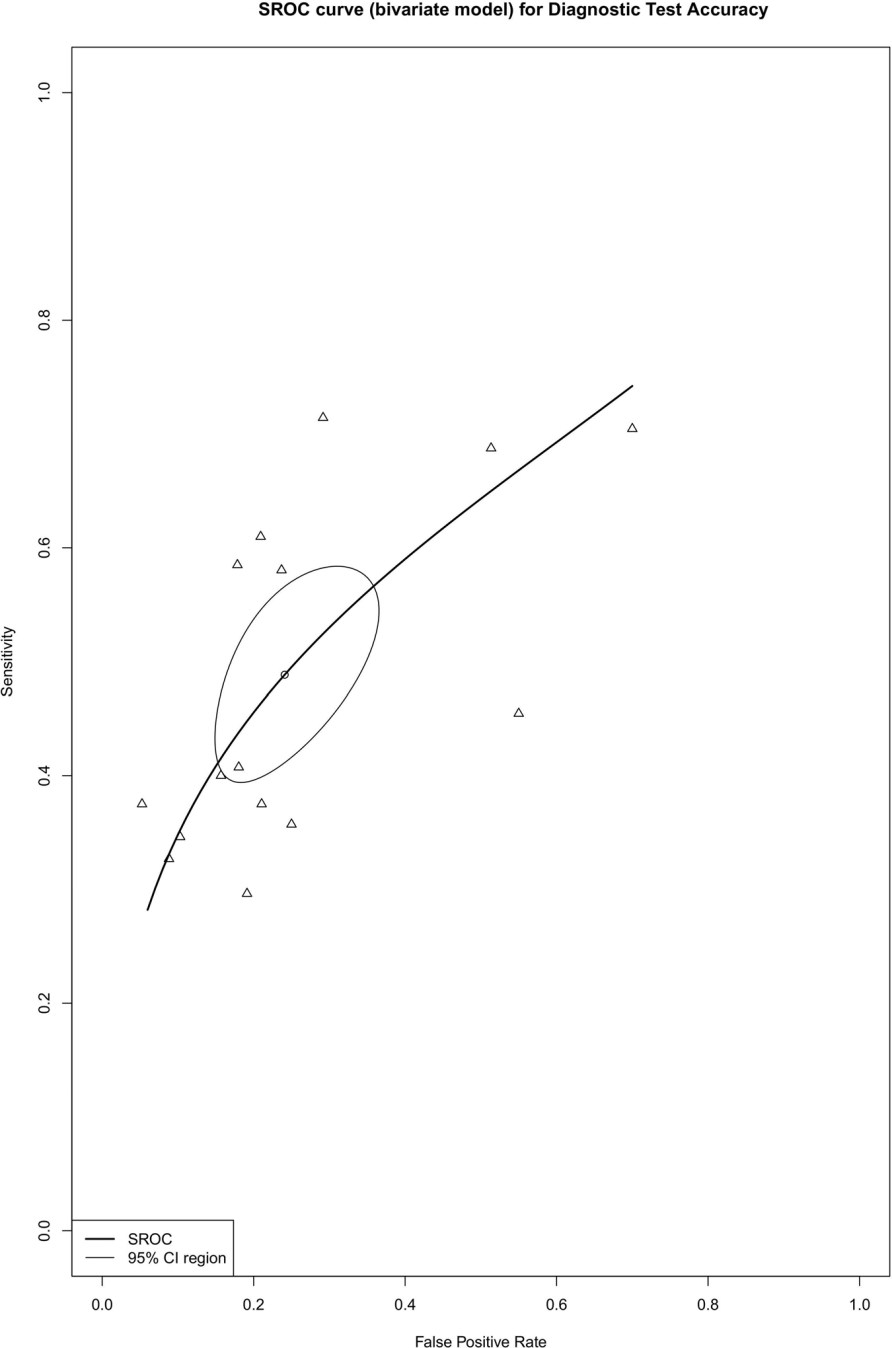
Bivariate summary estimates of sensitivity and specificity for all studies that evaluated the accuracy of detecting EMVI+. Sensitivity and specificity were plotted in receiver operating characteristic (ROC) space for individual studies. Hierarchical summary receiver operating characteristic (HSROC) curve was plotted through data points

The summary estimates for sensitivity and specificity for EMVI+ vs. EMVI− were 49% (95% CI, 41–56%) and 77% (95% CI, 67–84%), respectively. The DOR for detecting nodal involvement was 3.0 (95% CI, 2.0–4.4) (Fig. 6). The I^2^ value was 63% (95% CI, 35–79%) for sensitivity and 85% (95% CI, 76–90%) for specificity. Forest plots of sensitivity and specificity, along with their respective heterogeneity, are presented in Fig. S5.

In studies using a slice thickness of < 5 mm [12, 36, 39, 52, 61, 63, 66], the summary estimates for sensitivity and specificity for detecting EMVI+ were 48% (95% CI, 40–57%) and 75% (95% CI, 64–84%), respectively, with a DOR of 2.8 (95% CI, 1.8–4.4). The I^2^ value was 67% (95% CI, 39–92%) for sensitivity and 88% (95% CI, 80–92%) for specificity.

There were only two studies with a slice thickness of 5 mm or more [33, 53], which did not allow for a proper meta-analysis.

### Tumor deposits

Only one study evaluated the diagnostic accuracy of CT for tumor deposits in colon cancer [63], therefore we could not compute a meta-analysis on this category. They reported a sensitivity of 47% and specificity of 89%.

## Discussion

This systematic review and meta-analysis showed that CT can detect pT3–T4 colon tumors, with a pooled sensitivity of 83% and specificity of 70%. However, its accuracy declines when evaluating deeper tumor invasion beyond 5 mm into the muscularis propria (pT3cd–T4), locoregional nodal involvement (pN+), and the presence of EMVI.

Compared with the meta-analysis by Nerad et al [11], our results showed slightly lower sensitivity for pT3–T4 tumors (83% vs. 90%) and pT3cd–T4 tumors (67% vs. 77%), while demonstrating higher specificity for pT3cd–T4 (88% vs. 70%). Notably, both reviews highlight the limited diagnostic value of CT for nodal staging, with Nerad et al reporting higher sensitivity (71% vs. 64%) but identical specificity (67%). The higher sensitivity reported by Nerad et al across categories may stem from their smaller and less heterogeneous dataset.

Technical factors seemed to influence CT performance. Sensitivity generally improved with the use of thin-slice CT (< 5 mm) and MDCT, supporting the importance of optimized imaging protocols. Nevertheless, specificity remained limited, mainly because benign pericolic stranding often mimics tumor extension. Differentiating between inflammation and malignant spread is a well-recognized diagnostic challenge [11, 35]. Furthermore, retrospective studies showed higher accuracy than prospective studies, likely due to selection bias, clearer cases, and more complete data. The real-time data collection, outcome uncertainty, and broader case inclusion in prospective studies contribute to variability, potentially leading to slightly reduced sensitivity and specificity.

Despite the acceptable accuracy of CT to detect pT3-4 colon cancer, neoadjuvant therapy for this group of patients is not yet standard care. However, this paradigm is likely to shift in light of the FOxTROT trial, which demonstrated that preoperative chemotherapy in patients with locally advanced colon cancer led to significant tumor downstaging and fewer incomplete resections, without compromising perioperative safety [3]. This will require an integrated approach towards risk stratification, combining imaging data with molecular, tissue-based, blood-derived, and clinical information.

CT demonstrated only modest accuracy for nodal staging, with pooled sensitivity and specificity at 64% and 67%, respectively. This can be attributed to heterogeneous and inconsistent criteria for defining metastatic locoregional nodes, ranging from size-based thresholds to morphological assessments, as well as high interobserver variability [24, 64]. Given these limitations, CT-detected nodal involvement should not be relied upon solely to guide clinical decision-making.

While MRI-detected EMVI is an independent high-risk factor for adverse outcomes in rectal cancer, the role of CT-detected EMVI in colon cancer remains less well-established [68, 69]. Only a few studies in our review reported CT-detected EMVI and showed poor sensitivity. This is likely due to its subtle and variable imaging appearance, where features such as irregular vessel contours or peritumoral changes overlap with surrounding structures, leading to understaging [70]. Furthermore, interpretation of EMVI on CT is highly subjective, influenced by image quality, radiologist expertise, and the absence of standardized criteria. Tumor deposits, a recognized high-risk feature in rectal cancer, are poorly studied in colon cancer, with only one study in our review reporting their detection on CT. Further research is needed to clarify the prognostic value of CT-detected EMVI and tumor deposits in colon cancer, and to improve their radiological detection.

Our meta-analysis provides updated pooled estimates for CT-based staging in colon cancer and includes the most comprehensive evaluation to date of CT performance in detecting EMVI in colon cancer. However, considerable heterogeneity across studies was observed, likely driven by differences in CT acquisition protocols, radiologist training, and variable definitions of CT staging criteria. Furthermore, most included studies were conducted in controlled academic settings with carefully selected patient cohorts and high technical standards, which may limit applicability to routine clinical practice. This indicates the need for further validation in routine clinical settings, where variability in imaging quality, patient characteristics, and radiologist experience is even greater. The exclusion of studies lacking complete diagnostic data may also have introduced selection bias, emphasizing the importance of comprehensive and transparent reporting in primary studies. Publication bias is another concern, despite efforts to minimize it through a systematic literature search.

Future research should focus on prospective, multicentre studies conducted in real-world settings. Such studies should employ standardized CT protocols, establish clear and uniform criteria for high-risk features, and emphasize structured radiologist training. Moreover, advanced computational tools, such as artificial intelligence, hold promise for improving the detection of subtle features such as EMVI and tumor deposits [71]. Studies evaluating the clinical utility and cost-effectiveness of multimodality imaging strategies could support more individualized, risk-adapted staging approaches in colon cancer.

In conclusion, although CT shows high sensitivity for detecting pT3–4 tumors, its moderate specificity and limited accuracy for the detection of pT3cd-4, nodal involvement, and EMVI+ reduce its value as a standalone tool for guiding clinical decisions. These limitations indicate the need for standardized CT protocols, unified reporting criteria, and improved radiologist training to ensure staging consistency and reliability.

## Supplementary information

Supplementary information

## Abbreviations

DOR: Diagnostic odds ratio
EMVI: Extramural venous invasion
FN: False negative
FP: False positive
HSROC: Hierarchical summary receiver operating characteristic
NA: Not available
PRISMA-DTA: Preferred Reporting Items for Systematic Reviews and Meta-Analyses–Diagnostic Test Accuracy
QUADAS: Quality Assessment of Diagnostic Accuracy Studies
TN: True negative
TP: True positive
WE-MDCT: Water-enema multidetector CT
