# Supplementary material for: Diagnostic accuracy of CT for identifying high-risk colon cancer: a systematic review and meta-analysis
Source: Eur Radiol. 2025 Aug 16;36(2):1012–24. doi: 10.1007/s00330-025-11844-2 (PMC12953287; doi:10.1007/s00330-025-11844-2)
Supplement: Supplementary file 1 — Supplementary information [file 330_2025_11844_MOESM1_ESM.docx]

# Supplemental Content

## **Appendix S1** Literature Search

**Ovid MEDLINE**

| **#** | **Query** |
| --- | --- |
| 1 | "colorectal neoplasms"/ |
| 2 | exp "colonic neoplasms"/ |
| 3 | exp "Colorectal Neoplasms, Hereditary Nonpolyposis"/ |
| 4 | ((colon* or colorectal) adj7 (neoplasm* or cancer* or tumor* or tumor* or carcinoma* or adenocarcinoma*)).ti,ab,kf. |
| 5 | or/1-4 [colon cancer] |
| 6 | exp "neoplasm staging"/ |
| 7 | ((neoplasm* or cancer* or tumor* or tumor* or carcinoma* or adenocarcinoma*) adj3 (stag* or grading)).ti,ab,kf. |
| 8 | (TNM adj2 staging).ti,ab,kf. |
| 9 | or/6-8 [cancer staging] |
| 10 | exp "tomography, x ray computed"/ |
| 11 | "tomography x ray computed".ti,ab,kf. |
| 12 | "computed tomograph*".ti,ab,kf. |
| 13 | CT.ti,ab,kf. |
| 14 | (x-ray adj2 CAT).ti,ab,kf. |
| 15 | "virtual colonoscop*".ti,ab,kf. |
| 16 | or/10-15 [CT - all years] |
| 17 | limit 16 to dt=20150101-20220525 [CT - since 2015] |
| 18 | exp "extranodal extension"/ |
| 19 | ((extranodal or "extra-nodal" or extracapsular or "extra-capsular") adj2 (extension* or growth*)).ti,ab,kf. |
| 20 | "tumor deposit*".ti,ab,kf. |
| 21 | "tumor deposit*".ti,ab,kf. |
| 22 | or/18-21 [tumor deposits] |
| 23 | extramural.ti,ab,kf. |
| 24 | 'venous invasion'.ti,ab,kf. |
| 25 | 'vessel invasion'.ti,ab,kf. |
| 26 | EMVI.ti,ab,kf. |
| 27 | or/23-26 [EMVI] |
| 28 | 5 and 9 and (17 or 22 or 27) [colon cancer AND cancer staging AND (CT - since 2015 OR tumor deposits OR EMVI)] |

**Embase.com:**

| **#** | **Query** |
| --- | --- |
| #31 | #3 AND #7 AND (#15 OR #21 OR #28) AND ([conference abstract]/lim OR [conference review]/lim) |
| #30 | #3 AND #7 AND (#15 OR #21 OR #28) NOT ([conference abstract]/lim OR [conference review]/lim) |
| #29 | #3 AND #7 AND (#15 OR #21 OR #28) |
| #28 | #22 OR #23 OR #24 OR #25 OR #26 OR #27 |
| #27 | emvi:ti,ab,kw |
| #26 | 'vessel invasion':ti,ab,kw |
| #25 | 'venous invasion':ti,ab,kw |
| #24 | extramural:ti,ab,kw |
| #23 | 'extramural vessel invasion' |
| #22 | 'extramural venous invasion'/de |
| #21 | #16 OR #17 OR #18 OR #19 OR #20 |
| #20 | 'tumor deposit*':ti,ab,kw |
| #19 | 'tumor deposit*':ti,ab,kw |
| #18 | ((extranodal OR 'extra-nodal' OR extracapsular OR 'extra-capsular') NEAR/2 (extension* OR growth*)):ti,ab,kw |
| #17 | 'extranodal extension'/exp |
| #16 | 'tumor deposit'/exp |
| #15 | #14 AND [01-01-2015]/sd |
| #14 | #8 OR #9 OR #10 OR #11 OR #12 OR #13 |
| #13 | 'virtual colonoscop*':ti,ab,kw |
| #12 | ('x ray' NEAR/2 cat):ti,ab,kw |
| #11 | 'ct':ti,ab,kw |
| #10 | 'computed tomograph*':ti,ab,kw |
| #9 | 'tomography x ray computed':ti,ab,kw |
| #8 | 'x-ray computed tomography'/exp |
| #7 | #4 OR #5 OR #6 |
| #6 | (tnm NEAR/2 staging):ti,ab,kw |
| #5 | ((neoplasm* OR cancer* OR tumor* OR tumor* OR carcinoma* OR adenocarcinoma*) NEAR/3 (stag* OR grading)):ti,ab,kw |
| #4 | 'cancer staging'/exp |
| #3 | #1 OR #2 |
| #2 | ((colon* OR colorectal) NEAR/7 (neoplasm* OR cancer* OR tumor* OR tumor* OR carcinoma* OR adenocarcinoma*)):ti,ab,kw |
| #1 | 'colon cancer'/exp |

## **Table S1** Demographics and Study Characteristics

| Study | **Study characteristics** | | | | **Patient characteristics** | | | | ***Reason for Patient Exclusion*** |
| --- | --- | --- | --- | --- | --- | --- | --- | --- | --- |
|  | **Author** | **Year** | **Design** | **No. of Interpreters** | **No. of patients** | **No. of lesions** | **Age range (years)** | **Gender (male/female)** |  |
| 1 | Bedrikovetski et al.* | 2022 | Prospective | 2 (report and MDT) | 270 | 270 | Not separable from rectal | Not separable from rectal | No available imaging reports, or the reporting radiologist was also a member of the colorectal MDT |
| 2 | Choi et al. | 2015 | Retrospective | 3 | 64 | 64 | 68 | 26/38 | Metastatic disease on CT, rectal or appendiceal involvement, no accompanying imaging from referring institutions |
| 3 | Burton et al. | 2008 | Prospective | 2 | 33 | 33 | 72 (46-93) | 18/15 | Primary treatment was not surgical resection. |
| 4 | Dighe et al. | 2010 | Prospective | 2 | 84 | 84 | 75 (47–89) | NA | Rectal cancers, widespread metastases unsuitable for surgical resection, severe co-morbidities, small polyps not visible on CT |
| 5 | Olsen et al.* | 2020 | Retrospective | NA | 4832 | 4832 | 72 (21–99) | 2724/2502 | Missing data on T or N category, preoperative polypectomy, endoscopic resection only, clinical stage IV, neoadjuvant chemotherapy, synchronous tumors, palliative procedure with no tumor resection, palliative stent before resection, laparotomy without colon resection, misclassified rectal tumors as colon tumors |
| 6 | Sjovall et al. | 2015 | Retrospective | NA | 5624 | 5624 | 73 (19–98) | 2772 / 2852 | cTX or cNX stage, tumors discovered at autopsy |
| 7 | van de Weerd et al. | 2022 | Prospective | 5 | 44 | 44 | 70.5 (63–77) | 21 /23 | Rectal cancers, small sized colon tumor lesions which could not be visualized on CT, poor image quality, neoadjuvant therapy |
| 8 | Elzaki et al. | 2022 | Retrospective | 1 | 96 | 96 | 69 (47–74) | 47/49 | Rectal cancer, chemotherapy, neoadjuvant radiotherapy, distant metastases, not scanned preoperatively |
| 9 | Shekleton et al. | 2022 | Retrospective | 1 | 380 | 380 | 20-100 | 189/191 | Extended right hemicolectomy, non-curative surgery, no pre-operative scan, complete response to neo-adjuvant chemotherapy, histological T0 |
| 10 | Brouwer et al. | 2018 | Retrospective | 1 | 61066 | 61066 | 0-80+ | 31,690/30,715 | No lymph nodes resected, stage IV patients |
| 11 | Kim et al. | 2022 | Retrospective | 1 | 507 | 507 | 27–93 years (63.0 ± 11.6) | 243/264 | Previous other malignancy, previous abdominal surgery, and/or previous chemotherapy and/or radiotherapy before surgery, colon cancer identified on CT at the middle portion of the ascending colon or beyond, comorbidity on preoperative CT or medical status that could interfere with rLN assessment (intussusception, obstruction, abscess, liver cirrhosis), suboptimal lymph node assessment due to the presence of seeding lesions and/or extensive tumor infiltration on CT, poor image quality, MSI-low tumors |
| 12 | Rafaelsen et al. | 2022 | Prospective | 1 | 118 | 118 | 70.6 years (39–91) | 62/56 | Use of a pacemaker, implanted drug pumps or nerve stimulators, severe claustrophobia, prior radiotherapy/chemotherapy, or any other condition or disease as assessed by the investigator made it inappropriate for the patient to participate in the trial. |
| 13 | Hunter et al. | 2017 | Prospective | 2 | 53 | 53 | 69.3 (SD +/- 13.6) | 34/19 | Reasons given only for MRI, not for CT. |
| 14 | Cárcamo G. et al. | 2022 | Prospective | 2 | 47 | 47 | NA | NA | Emergency surgery, non-oncological resections, adenocarcinoma limited to a polyp, neoadjuvant treatment, hereditary colorectal diseases. |
| 15 | Shkurti et al. | 2023 | Retrospective | NA | 39,565 | 39,565 | 71 (18–99) | 19,948/19,617 | Presence of metastases, neoadjuvant treatment, previous colorectal cancer, incomplete or unknown staging. |
| 16 | Park et al. | 2019 | Prospectively registered, retrospectively reviewed | 2 | 38 | 38 | 25–78 (mean 59.5 ± 12.6) | 20/18 | Technical failure due to uncontrolled respiration, medullary carcinoma per histopathology |
| 17 | Wiegering et al. | 2015 | Retrospective | 2 | 210 | 210 | 69, 94 (27.3–92.5) | NA | Patients with two cancers, a previous colon resection, or MRI |
| 18 | Malmstrøm et al. | 2017 | Prospective | 1 | 35 | 35 | Mean 68, Median 70, Range 51–89 | 28/7 | Prior radio chemotherapy, prior endoscopic resection or attempted endoscopic resection of the tumor, pregnancy, NArmed consent, severe coagulopathy and/or disseminated disease, metastatic disease, stented, neoadjuvant treatment, no consent, other reasons. |
| 19 | Santiago et al. | 2016 | Prospective | 3 | 74 | 74 | Median 73 (45–89) | 43/31 | Inadequate acquisition technique, neoadjuvant therapy for synchronous rectal cancer, loss to follow-up, oncologic death, irresectability recognised intraoperatively, incurable disseminated disease, death due to comorbidities |
| 20 | Fernandez et al. | 2019 | Retrospective | 3 | 150 | 150 | Median 70.2 years (39–94) | 79/71 | No available preoperative CT scan |
| 21 | Hong et al. | 2022 | Retrospective | 3 | 292 | 292 | Median 65 (29-89) | 153/139 | Neoadjuvant therapy, no appropriate pathologic data, multiple tumors, tumors not visible on CT, poor quality of pre-operative imaging. |
| 22 | Sasaki et al. | 2017 | Retrospective | 1 | 370 | 380 | 69 (24–93) | 240/130 | Synchronous extra-colorectal malignancy, associated inflammatory disease, preoperative treatment |
| 23 | Liu et al. | 2021 | Retrospective | 2 | 216 | 216 | median 61 (31-83) | 135/81 | Comorbidity or history of other malignancies, more than one synchronous colonic tumor, neoadjuvant therapy, no preoperative abdominal or pelvic CT, surgery beyond 14 days after contrast-enhanced CT. |
| 24 | Hong et al. | 2020 | Retrospective | 4 | 88 | 88 | 72 (34–93) | NA | Rectal cancers, mucinous cancers, colon tumor lesion not visualized on CT, poor image quality, neoadjuvant chemotherapy. |
| 25 | Rollvén et al. | 2019 | Retrospective | 2 | 94 | 94 | Median 72 (45–90) | 49/45 | CT examination not fulfilling the standards stated in the study protocol (no intravenous contrast or different CT scanner), metastatic disease. |
| 26 | Sikkenk et al. | 2023 | Retrospective | NA | 31,637 | 31,637 | 57.2% was >=70 years | 16,551/15,086 | Synchronous tumors of the colon, local excision, “watch and wait” strategy, emergency operation, neoadjuvant therapy |
| 27 | Liu et al. | 2019 | Retrospective | 2 | 116 | 116 | Mean 60.9 ± 9.9 (36-83) | 78/38 | Other history of malignancy, neoadjuvant therapy, mucinous tumor, no pre-operative CT scans, surgery beyond one month after imaging acquisition, insufficient quality of key images for staging tumor. |
| 28 | Park et al. | 2020 | Retrospective | 5 | 110 | 110 | Median 67 (26–90) | 58/52 | Insufficient information on histopathology report, neoadjuvant chemotherapy, intussusception due to colon cancer |
| 29 | Rollvén et al. | 2013 | Retrospective | 2 | 28 | 27 | 73 (41-86) | 17/11 | No patient consent, general poor condition of the patient, metal objects preventing MRI, no surgery because of extensive metastatic disease, preoperative chemotherapy, death before operation, final diagnosis of metastases |
| 30 | McAvoy et al. | 2018 | Retrospective | NA | 53 | 53 | Median 72 (46–93) | 35/18 | Rectal and colonic tumors presenting as an emergency without complete preoperative CT imaging |
| 31 | Wang et al. | 2023 | Prospective | 4 | 190 | 190 | Mean 64 (30-93) | 86/104 | Emergency setting, contraindications to contrast-agent, severe systemic disease, inappropriate for elective surgery without neoadjuvant therapy. |
| 32 | Norgaard et al. | 2014 | Retrospective | 1 | 74 | 74 | Mean 72 (32-90) | 29/45 | Tumors that could not be identified on CT, inoperable patients, two tumors in the sigmoid colon located close to each other. |
| 33 | Malmstrom et al. | 2017 | Retospective | 5 in total (combined) | 501 | 501 | Mean 69,4 | 271/230 | Missing radiological staging: urgent CT scans, preoperative chemotherapy, stented, invagination, other (unknown) reasons. |
| 34 | Platt et al. | 2023 | Retrospective | NA | 57 | 57 | 73 (45-90) | 27/30 | Non-colonic malignancy (including rectal cancer), synchronous CC, histological subtype other than adenocarcinoma or mucinous adenocarcinoma, active appendicitis, diverticulitis, colitis or perforation when undergoing radiological staging, surgery performed with only palliative intent, prior neoadjuvant treatment, or radiological staging, pathological staging or MMR status were not accessible. |
| 35 | Erbs et al. | 2020 | Retrospective | 1 | 590 | 590 | dMMR: mean 73,8; pMMR: mean 70,4 | dMMR: 37/98; pMMR: 249/206 | Neoadjuvant treatment, no preoperative CT scan, unknown M category, unknown MMR status, insufficient patient data. |
| 36 | Pedersen et al. | 2021 | Retrospective | 1 | 6102 | 6102 | Median 72, (23-97) | 3161/2941 | No formal bowel resection, previous emergent procedure, local resection of a malignant polyp, information on synchronous tumors was either missing or affirmative, pathologically rectal tumors. |
| 37 | Elibol et al. | 2016 | Retrospective | 2 | 141 | 141 | Mean 66.7±11.67 (24–92) | 83/58 | Rectal or rectosigmoid tumors, CT or histopathologic evaluation performed at another hospital, inoperable patients, and postoperative histopathologic diagnosis not revealing carcinoma. |
| 38 | Ippolito et al. | 2023 | Retrospective | 3 | 75 | 75 | Mean 80 (±11) | 32/43 | Contraindication to iodinated contrast agents, incomplete CT protocol (i.e., unenhanced sequence only), staging CECT performed in other institution(s), CECT acquired post-operative exclusively. |
| 39 | Korsbakke et al. | 2019 | Retrospective | 1 | 354 | 354 | 75 (33 - 95) | 175/179 | Synchronous tumors, non-resectional surgery, neoadjuvant treatment, and/or mising explicit stage according to TNM classification |
| 40 | Mou et al. | 2021 | Retrospective | 2 | 106 | 106 | Mean 62.93 (18 – 83) | 57/49 | No preoperative enhancement CT scan, poor quality of CT images, no visible tumor on CT images, preoperative chemotherapy or radiotherapy. |
| 41 | Venara et al. | 2015 | Retrospective | 2 | 71 | 76 | Mean 73.1 (45 – 95) | 42/29 | No surgery, no preoperative WE-MDCT, rectal cancer. |
| 42 | Hernández et al. | 2023 | Retrospective | 1 | 1950 | 1950 | 70.2 (SD 12.3) | 1115/835 | <18 years old, inability to achieve a whole tumor resection or palliative surgery (R2), no preoperative CT scan, no pathological adenocarcinoma, missing information. |
| 43 | Karahacioglu et al. | 2024 | Retrospective | 2 | 137 | 137 | 65.9 (35–95) | 82/55 | rectosigmoid junction tumors, small lesions not detectable on CT, suboptimal/non diagnostic CT scans, synchronous multiple colonic tumors, history of neoadjuvant therapy |
| 44 | Leonhardi et al. | 2024 | Retrospective | 2 | 108 | 108 | mean age of 70.08 ± 14.34 | 59/49 | NArmation |
| 45 | van den Berg et al. | 2023 | Retrospective | 1 | 176 | 176 | mean 71.9 SD 9.5 | 95/81 | metastatic disease, ongoing palliative chemotherapy at the time of surgery, history of abdominal radiotherapy, second primary tumor of benign or malignant origin |
| 46 | Bompou et al. | 2024 | Prospective | 2 | 120 | 120 | 76 (16) | 81/39 | allergy or contraindication to receive intravenous contrast, claustrophobia, incompatible implants with MRI, active sepsis, untreated physical or mental disability, lack of compliance with the research protocol and, non granting of signed informed consent. |

*The results for age range and gender are not separable from patients with rectal cancer

WE-MDCT – water-enema multidetector computed tomography; MDT – multidisciplinary team; MMR – mismatch repair; NA – not available

## **Table S2** Technical Characteristics of Studies Analyzed

| Study | **Study characteristics** | | | | **Imaging characteristics** | | | | **Bowel preparation** | **Colon insufflation** | |
| --- | --- | --- | --- | --- | --- | --- | --- | --- | --- | --- | --- |
|  | **Author** | **Year** | **Interpreter blinded** | **Pathologist blinded** | **Imaging technique** | **CT Slice Thickness (mm)** | **IV contrast** | **Oral contrast** |  | **Air** | **Water** |
| 1 | Bedrikovetski et al. | 2022 | Yes | NA | Abdominopelvic CT | NA | Yes, unspecified | Yes, unspecified | NA | NA | Yes |
| 2 | Choi et al. | 2015 | Yes | NA | CT | NA | NA | NA | NA | NA | NA |
| 3 | Burton et al. | 2008 | Yes | NA | Single slice spiral CT | 10 | Iohexol 300, at 3 ml s-1 | 10 ml gastrografin in 990 ml water | 10 ml gastrografin in 300 ml of tap water orally the night before | NA | NA |
| 4 | Dighe et al. | 2010 | Yes | NA | 16-slice and 64-slice MDCT | 1·5 | 100 ml Ultravist 0300 at 3 ml/s | 1 litre of water 30–45 min before the scan | No | NA | NA |
| 5 | Olsen et al. | 2020 | Yes | NA | MDCT | 5 in most patients and 5 in the remainder | NA | NA | NA | NA | NA |
| 6 | Sjovall et al. | 2015 | Yes | NA | NA | NA | NA | NA | NA | NA | NA |
| 7 | van de Weerd et al. | 2022 | Yes | NA | 16-channel CT | 3 | 90–150 ml Iodine-based at 3–5 ml/s, followed by bolus injection of 30 cc normal saline | No | No | NA | NA |
| 8 | Elzaki et al. | 2022 | Yes | NA | CT | 5, 3, 2 | NA | NA | NA | NA | NA |
| 9 | Shekleton et al. | 2022 | Yes | NA | CT | 1.5 | NA | NA | NA | NA | NA |
| 10 | Brouwer et al. | 2018 | Yes | NA | NA | NA | NA | NA | NA | NA | NA |
| 11 | Kim et al. | 2022 | Yes | NA | MDCT 40 detector and 64 detector | 3-3.5 | 120 mL non-ionic iodinated contrast material at 3–4 mL/s | NA | NA | NA | NA |
| 12 | Rafaelsen et al. | 2022 | Yes | NA | Contrast-enhanced CT 64-slice | 3 | 100 mL iomeprol 300 mg I/mL at 4 mL/sec | NA | NA | NA | NA |
| 13 | Hunter et al. | 2017 | Yes | NA | CT | 8 or 5 | Yes, unspecified | NA | No | NA | NA |
| 14 | Cárcamo G. et al. | 2022 | Yes | NA | MDCT | 0.625 | 100 ml of ioversol 68% 320 mg/ml at 3 ml/s | NA | NA | NA | NA |
| 15 | Shkurti et al. | 2023 | Yes | NA | MDCT | NA | NA | NA | NA | NA | NA |
| 16 | Park et al. | 2019 | Yes | NA | MDCT | 2–2.5 | 90–95 mL intravenous injection of nonionic contrast media Iomeron 300 at 1.2–1.5 mL/s | NA | NA | NA | NA |
| 17 | Wiegering et al. | 2015 | Yes | NA | MDCT | 1 and 3 | power injection of 90–110 ml Imeron® at an iodine concentration of 300 mg/ml at 3 ml/s | 1 l water with 30 ml of Gastrolux® CT 370 mg/ml over 45 min before the scan | 500–800 ml water rectal enema with Gastrolux® | NA | NA |
| 18 | Malmstrøm et al. | 2017 | NA | NA | 64-slice and 128-slice MDCT | NA | NA | NA | NA | NA | NA |
| 19 | Santiago et al. | 2016 | Yes | Yes | Optimized thoraco-abdomino-pelvic MDCT | 1.25 | Iodinated contrast (Ultravist 300 mg/I per mL, 2 mL/Kg of patient weight, up to a maximum of 140 mL) at 2.5 mL/s, with a delay of 55 s for thorax and 70 s for abdomen and pelvis | 400 mL water 20 min before and 400 mL water immediately before the acquisition | No | Yes | Yes |
| 20 | Fernandez et al. | 2019 | Yes | NA | NA | NA | NA | NA | NA | NA | NA |
| 21 | Hong et al. | 2022 | Yes | NA | 16–320 slice CT | 1-5 | 90–150 ml iodine-based contrast at 3—5 ml/second, followed by normal saline bolus injection | 20 cc Omnipaque 300 mg/ml in 1 L of water, 1 h before the examination | No | NA | NA |
| 22 | Sasaki et al. | 2017 | Yes | NA | contrast-enhanced CT (ceCT) | NA | Iopamidol 300 at 2.0 ml/s | NA | NA | NA | NA |
| 23 | Liu et al. | 2021 | Yes | NA | MDCT scanners | 3 | bolus intravenous injection of 1.5 ml/kg of non-ionic contrast agent Ultravist 350 followed by 30 ml saline flush at 3 ml/s | NA | Fasting more than 4 hours and ingesting 600-800 ml tap water before scanning | NA | Yes |
| 24 | Hong et al. | 2020 | Yes | NA | 16–64 slice CT | 1-2 | 90–130 ml iodine-based intravenous contrast at 3 ml/s, followed by bolus injection of 30 cc normal saline in portal-venous phase at 70 s delay | 20 cc omnipaque 300 mg/ml | No | NA | NA |
| 25 | Rollvén et al. | 2019 | Yes | NA | 64-channel multislice CT | 5 with 1 overlap (interval 4) with the original 0.625 | 300 mg I/ml, Iomeron, Bracco, < 60 kg body weight 120 ml, > 60 kg body weight 150 ml) in portal-venous phase (delay 90 s after injection) | No | No | NA | NA |
| 26 | Sikkenk et al. | 2023 | Yes | NA | MDCT | NA | NA | NA | NA | NA | NA |
| 27 | Liu et al. | 2019 | Yes | NA | MDCT | 3 | bolus intravenous injection of 1.5 ml/kg of non-ionic contrast agent Ultravist 350 followed by 30 ml saline flush at 3 ml/s | NA | Low-residue diet for two days and fasting for 4−5 h before CT | NA | 600−800 ml tap water ingested at 20 min prior to examination. |
| 28 | Park et al. | 2020 | Yes | Yes | MDCT | 2-2.5 | 90–95-mL intravenous injection of nonionic contrast media Iomeron® 300 at 1.2–1.5 mL/s | NA | NA | NA | NA |
| 29 | Rollvén et al. | 2013 | Yes | NA | from single slice technology up to 64-slice helical CT | 5-8 | Yes, not specified | NA | NA | NA | NA |
| 30 | McAvoy et al. | 2018 | Yes | NA | MDCT (64 slice) | 2.5 | Iodine-based contrast agent | NA | NA | NA | NA |
| 31 | Wang et al. | 2023 | Yes | NA | MDCT | 3 | Bolus intravenous injection of 1.5 ml/kg of non-ionic contrast agent Ultravist 350 followed by 30 ml saline flush at 3 ml/s | NA | Fasting more than 4 hours and ingesting 600-800 ml tap water before scanning | NA | Ingesting 600-800 ml tap water before scanning. |
| 32 | Norgaard et al. | 2014 | Yes | NA | 64-slice CT | NA | 100 ml iomeprol 300 mg I/ml at 4 ml/sec | NA | NA | NA | NA |
| 33 | Malmstrom et al. | 2017 | Yes | NA | 64-slice CT | NA | 100 mL Iomeron® 350 mg/mL at 3 mL/s | 1 L water 15 min prior to the scan | No | NA | NA |
| 34 | Platt et al. | 2023 | Yes | NA | 128 detector CT | MaX 2.5  axial and 3  coronal reconstructed | 350 mg ml−1 iodinated contrast and 65 s delay | NA | NA | NA | NA |
| 35 | Erbs et al. | 2020 | Yes | Yes | 64-channel MDCT | NA | 100 ml iomeprol | NA | NA | NA | NA |
| 36 | Pedersen et al. | 2021 | NA | NA | NA | NA | NA | NA | NA | NA | NA |
| 37 | Elibol et al. | 2016 | NA | NA | 16 or 64-slice MDCT | 1-2 | 100mL water soluble nonionic contrast agent | Oral positive and negative contrast agent | Rectal contrast agent | NA | NA |
| 38 | Ippolito et al. | 2023 | Yes | NA | Contrast-enhanced CT (ceCT) | NA | NA | NA | NA | NA | NA |
| 39 | Korsbakke et al. | 2019 | No (based on clinical data) | No (based on clinical data) | SOMATOM Definition Flash (Siemens, Munich, Germany) or Quilion ONE (Canon, Zoetermer, the Netherlands) | 1,2 or 5 | NA | NA | NA | NA | NA |
| 40 | Mou et al. | 2021 | Yes | NA | contrast 64-section MDCT | 8 | Ultravist 300 Iopamidol | No | 1L water and 10mg of butylscopolamine bromide | NA | No |
| 41 | Venara et al. | 2015 | Yes | NA | WE-MDCT; 16-row MDCT | NA | 2ml/kg iodine 350 g/L at 3,5 mL | No | Colon was filled with | NA | Yes |
| 42 | Hernández et al. | 2023 | Yes | NA | NA | NA | NA | NA | NA | NA | NA |
| 43 | Karahacioglu et al. | 2024 | Yes | NA | 64 or 128 × 2-slice MDCT scanners (Siemens Somatom Definiton AS, Siemens Somatom Definition Flash) | 3, MPR 1 mm | 90–150 ml iodine at 3–5 ml/s, followed by bolus 30 cc normal saline | 50 cc Urografin diluted in 1.5 l of water 1.5 h before the examination | nil per os for 4–6 h before the examination | NA | NA |
| 44 | Leonhardi et al. | 2024 | Yes | NA | 128-slice CT scanner (Ingenuity 128, Philips, Hamburg, Germany) | 1 | 90 mL iodine-based contrast (Imeron 400 MCT) at 2–4.0 mL/s | NA | NA | NA | NA |
| 45 | van den Berg et al. | 2023 | Yes | NA | Brilliance iCT 256slice or a Brilliance 64 CT scanner | 1 | 100 mL intravenous contrast (Iomeron, iomeprol300 mg I/mL) | NA | NA | NA | NA |
| 46 | Bompou et al. | 2024 | Yes | NA | 128 row MDCT scanner | 1 | Dynamic contrast medium | NA | NA | NA | NA |

MDCT – multidetector computed tomography; NA – not available

## **Figure S1** Quality assessment of diagnostic accuracy studies (QUADAS)–2 scoring list for included studies.

| **Study** | **Risk of bias** | | | | **Applicability concerns** | | |
| --- | --- | --- | --- | --- | --- | --- | --- |
|  | **Patient election** | **Index test** | **Reference standard** | **Flow and timing** | **Patient selection** | **Index test** | **Reference standard** |
| Bedrikovetski et al. | ☺ | ☺ | ☺ | ☹ | ☺ | ☺ | ☺ |
| Choi et al. | ? | ☺ | ☺ | ☺ | ☺ | ☺ | ☺ |
| Burton et al. | ☺ | ☺ | ☺ | ☺ | ☺ | ☺ | ☺ |
| Dighe et al. | ☺ | ☺ | ☺ | ☺ | ☺ | ☺ | ☺ |
| Olsen et al. | ☹ | ☺ | ☺ | ☹ | ☺ | ☺ | ☺ |
| Sjovall et al. | ☺ | ☺ | ☺ | ☹ | ☺ | ☺ | ☺ |
| van de Weerd et al. | ☺ | ☺ | ☺ | ☹ | ☺ | ☺ | ☺ |
| Elzaki et al. | ? | ☺ | ☺ | ☹ | ☺ | ☺ | ☺ |
| Shekleton et al. | ? | ☺ | ☺ | ☺ | ☺ | ☺ | ☺ |
| Brouwer et al. | ☹ | ☺ | ☺ | ☺ | ☺ | ☺ | ☺ |
| Kim et al. | ☹ | ☺ | ☺ | ☺ | ☺ | ☺ | ☺ |
| Rafaelsen et al. | ? | ☺ | ☺ | ☹ | ☺ | ☺ | ☺ |
| Hunter et al. | ☺ | ☺ | ☺ | ☹ | ☺ | ☺ | ☺ |
| Cárcamo G. et al. | ☺ | ☺ | ☺ | ☺ | ☺ | ☺ | ☺ |
| Park et al. | ? | ☺ | ☺ | ☺ | ☺ | ☺ | ☺ |
| Wiegering et al. | ? | ☺ | ☺ | ☺ | ☺ | ☺ | ☺ |
| Malmstrøm et al. | ☹ | ☺ | ☺ | ☹ | ☺ | ☺ | ☺ |
| Santiago et al. | ☹ | ☺ | ☺ | ☹ | ☺ | ☺ | ☺ |
| Fernandez et al. | ☺ | ☺ | ☺ | ☺ | ☺ | ☺ | ☺ |
| Sasaki et al. | ☺ | ☺ | ☺ | ☺ | ☺ | ☺ | ☺ |
| Liu et al. | ☺ | ☺ | ☺ | ☺ | ☺ | ☺ | ☺ |
| Hong et al. (2020) | ☹ | ☺ | ☺ | ☺ | ☺ | ☺ | ☺ |
| Rollvén et al. (2019) | ☺ | ☺ | ☺ | ☹ | ☺ | ☺ | ☺ |
| Liu et al. | ☹ | ☺ | ☺ | ☺ | ☺ | ☺ | ☺ |
| Park et al. | ☺ | ☺ | ☺ | ☹ | ☺ | ☺ | ☺ |
| Rollvén et al. (2013) | ☹ | ☺ | ☺ | ☹ | ☺ | ☺ | ☺ |
| McAvoy et al. | ☺ | ☺ | ☺ | ☺ | ☺ | ☺ | ☺ |
| Wang et al. | ☹ | ☺ | ☺ | ☹ | ☺ | ☺ | ☺ |
| Norgaard et al | ☺ | ☺ | ☺ | ☺ | ☺ | ☺ | ☺ |
| Malmstrom et al. | ☺ | ☺ | ☺ | ☹ | ☺ | ☺ | ☺ |
| Erbs et al | ☺ | ☺ | ☺ | ☹ | ☺ | ☺ | ☺ |
| Pedersen et al. | ☺ | ☺ | ☺ | ☺ | ☺ | ☺ | ☺ |
| Elibol et al. | ☺ | ☺ | ☺ | ☺ | ☺ | ☺ | ☺ |
| Korsbakke et al. | ? | ☺ | ☺ | ☹ | ☺ | ☺ | ☺ |
| Mou et al. | ☺ | ☺ | ☺ | ☺ | ☺ | ☺ | ☺ |
| Venara et al. | ☺ | ☺ | ☺ | ☹ | ☺ | ☺ | ☺ |
| Hong et al. (2022) | ? | ☺ | ☺ | ☹ | ☺ | ☺ | ☺ |
| Ippolito et al. | ☹ | ☺ | ☺ | ☺ | ☺ | ☺ | ☺ |
| Platt et al. | ☹ | ☺ | ☺ | ☹ | ☺ | ☺ | ☺ |
| Shkurti et al. | ☺ | ☺ | ☺ | ☹ | ☺ | ☺ | ☺ |
| Sikkenk et al. | ? | ☺ | ☺ | ☹ | ☺ | ☺ | ☺ |
| Hernández et al. | ☺ | ☺ | ☺ | ☺ | ☺ | ☺ | ☺ |
| Karahacioglu et al. | ☺ | ☺ | ☺ | ☺ | ☺ | ☺ | ☺ |
| Leonhardi et al. | ? | ☺ | ☺ | ☺ | ☺ | ☺ | ☺ |
| van den Berg et al. | ☺ | ☺ | ☺ | ☹ | ☺ | ☺ | ☺ |
| Bompou et al. | ☹ | ☺ | ☺ | ☹ | ☺ | ☺ | ☺ |

☺ Low Risk ☹ High Risk ? Unclear Risk

## **Figure S2** Forest plot depicting sensitivity (A) and specificity (B) for differentiating T1–T2 versus T3–T4 tumors

1. Sensitivity


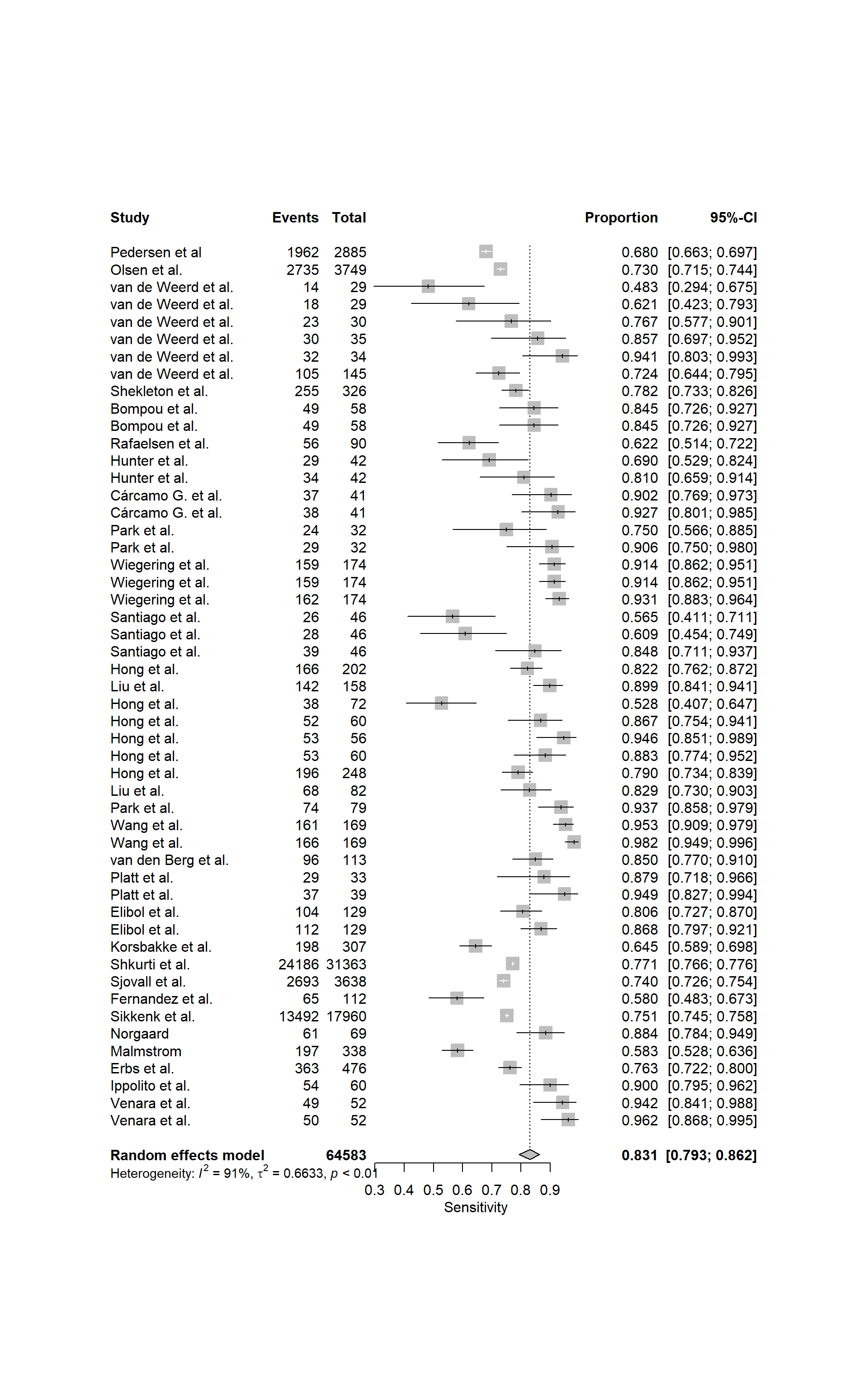


*’Events’ represent TP; ‘Total’ represents TP + FN*

1. Specificity


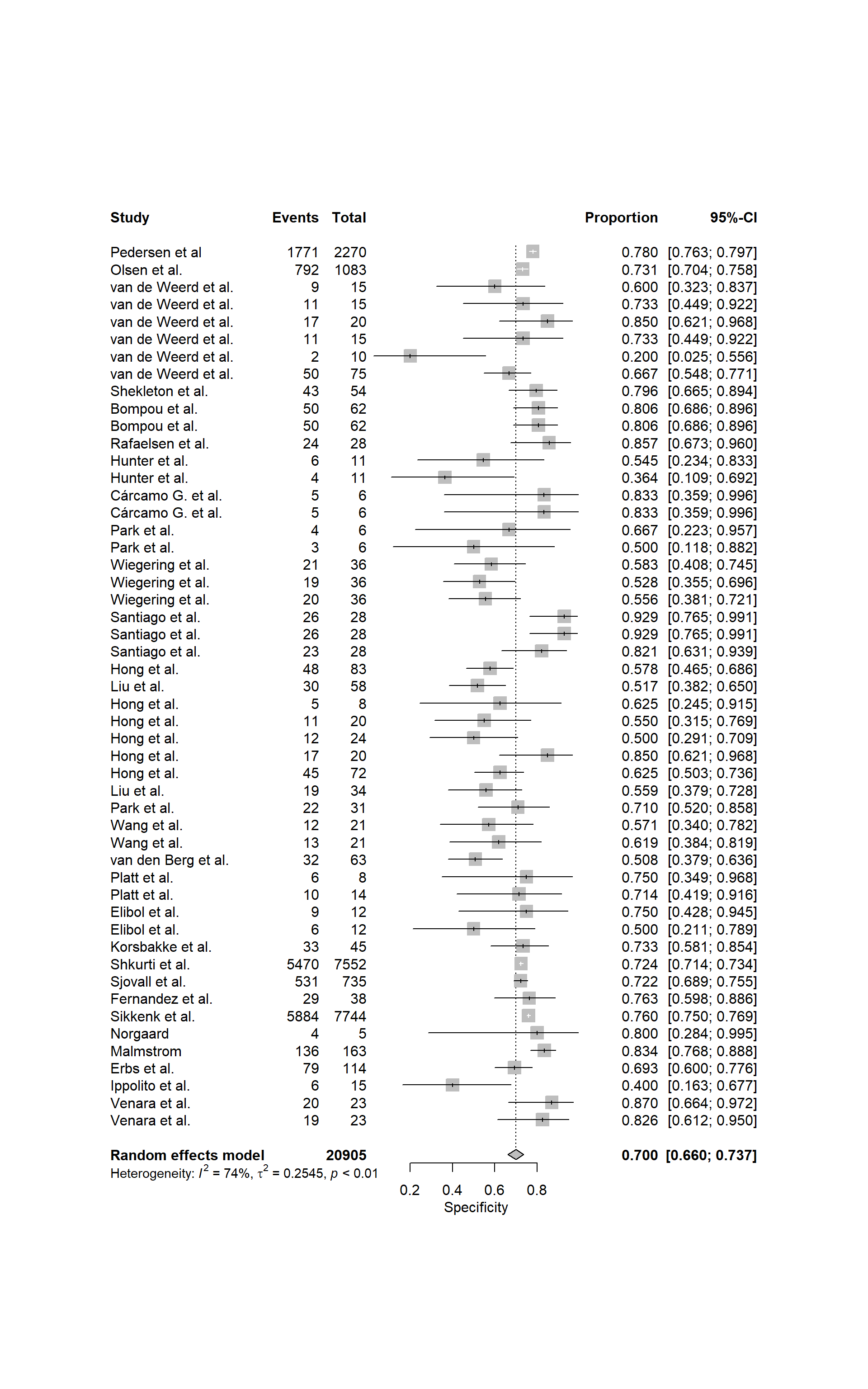


*’Events’ represent TN; ‘Total’ represents TN + FP*

## **Figure S3**: Forest plot depicting sensitivity (A) and specificity (B) for differentiating T1–T3ab versus T3cd–T4

1. Sensitivity


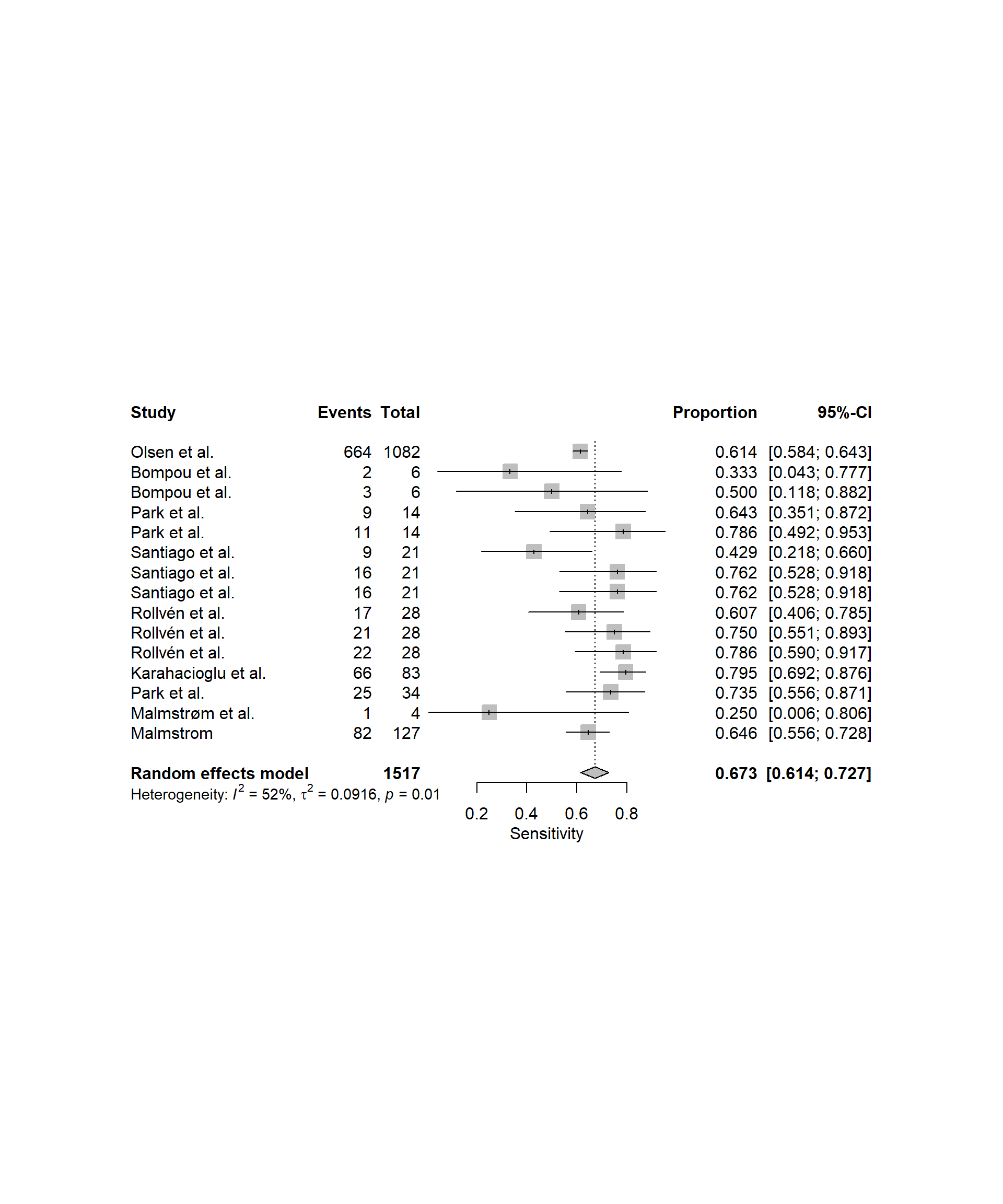


*’Events’ represent TP; ‘Total’ represents TP + FN*

1. Specificity


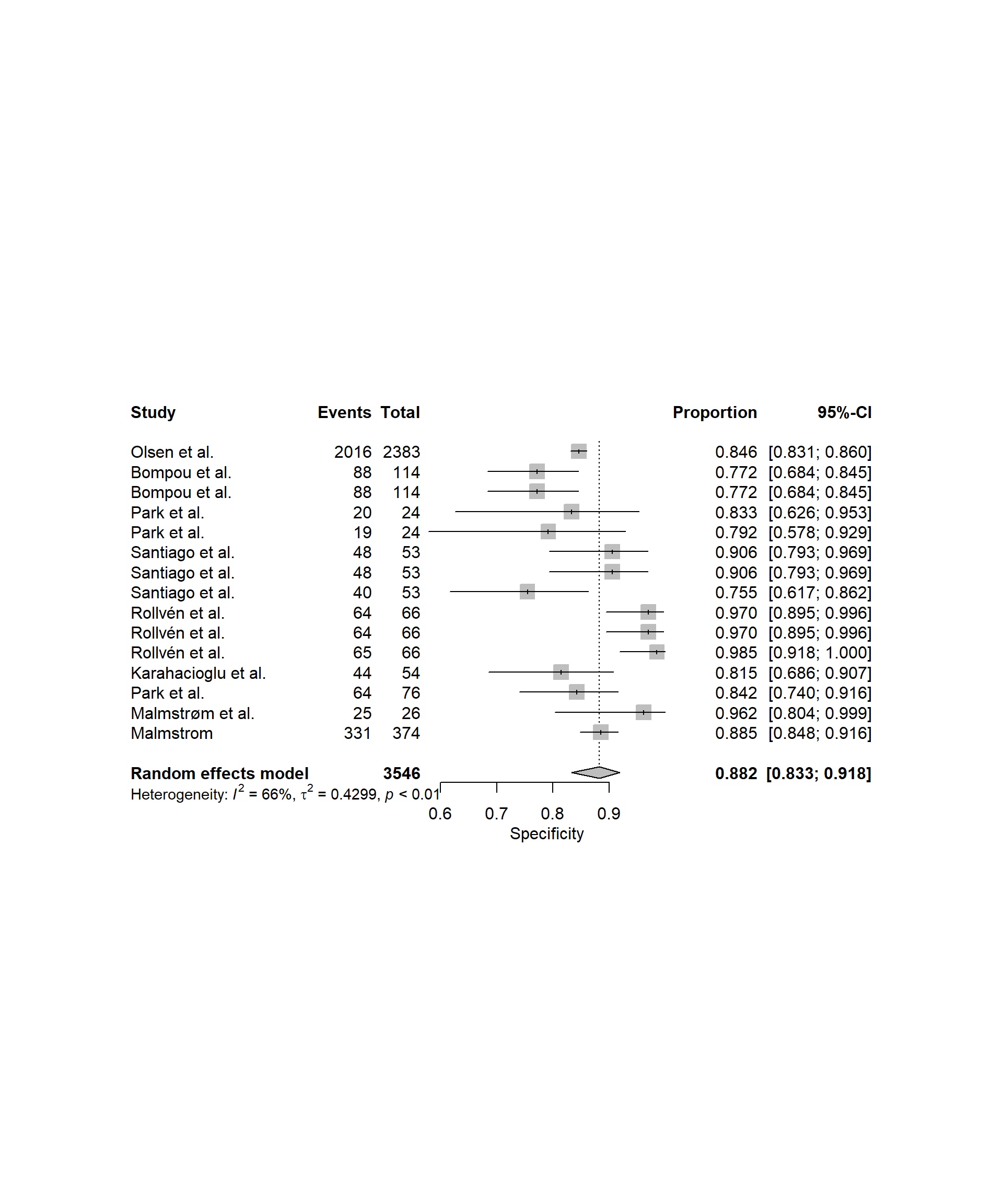


*’Events’ represent TN; ‘Total’ represents TN + FP*

## **Figure S4** Forest plot depicting sensitivity (A) and specificity (B) for differentiating N0 Versus N+

1. Sensitivity


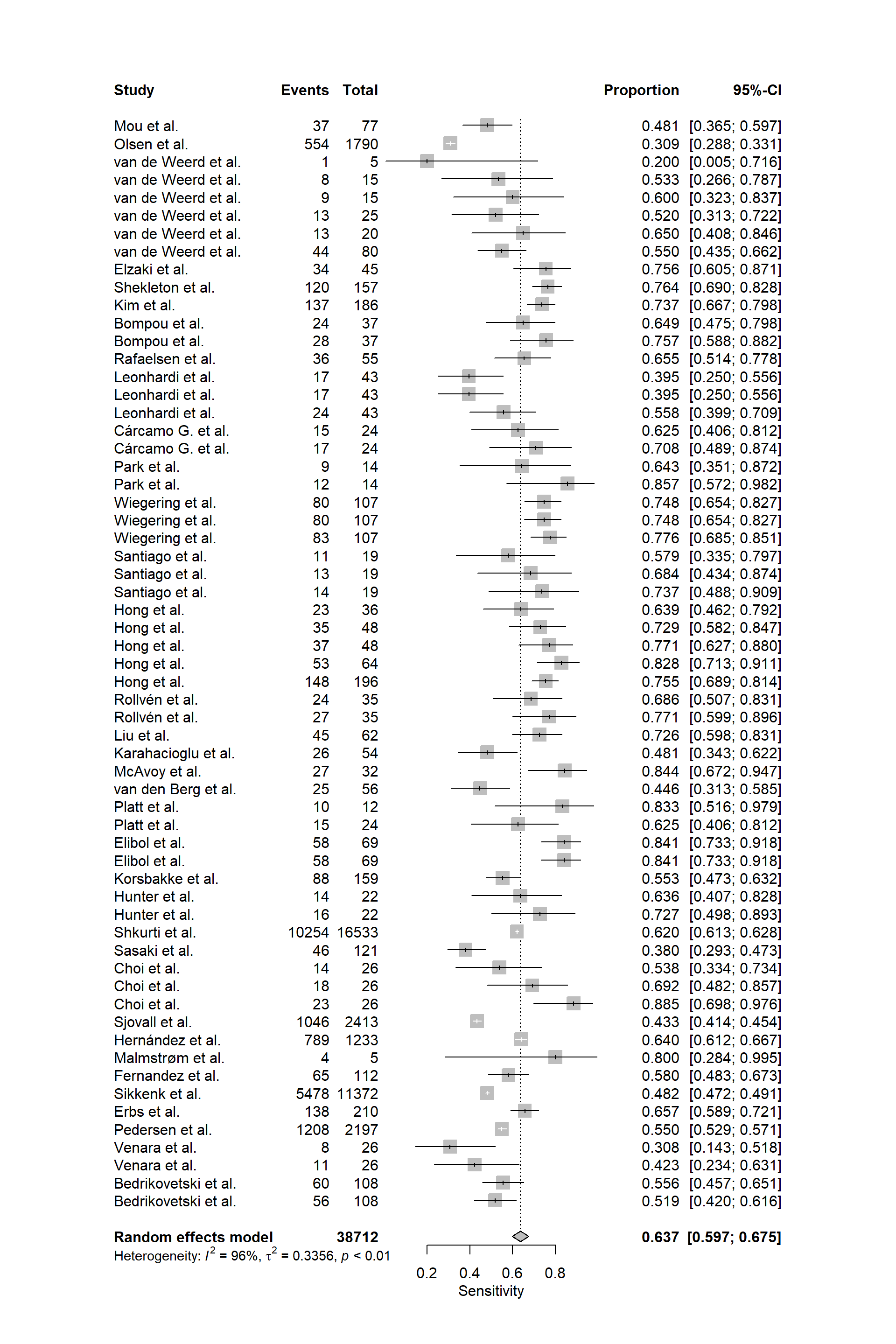


*’Events’ represent TP; ‘Total’ represents TP + FN*

1. Specificity


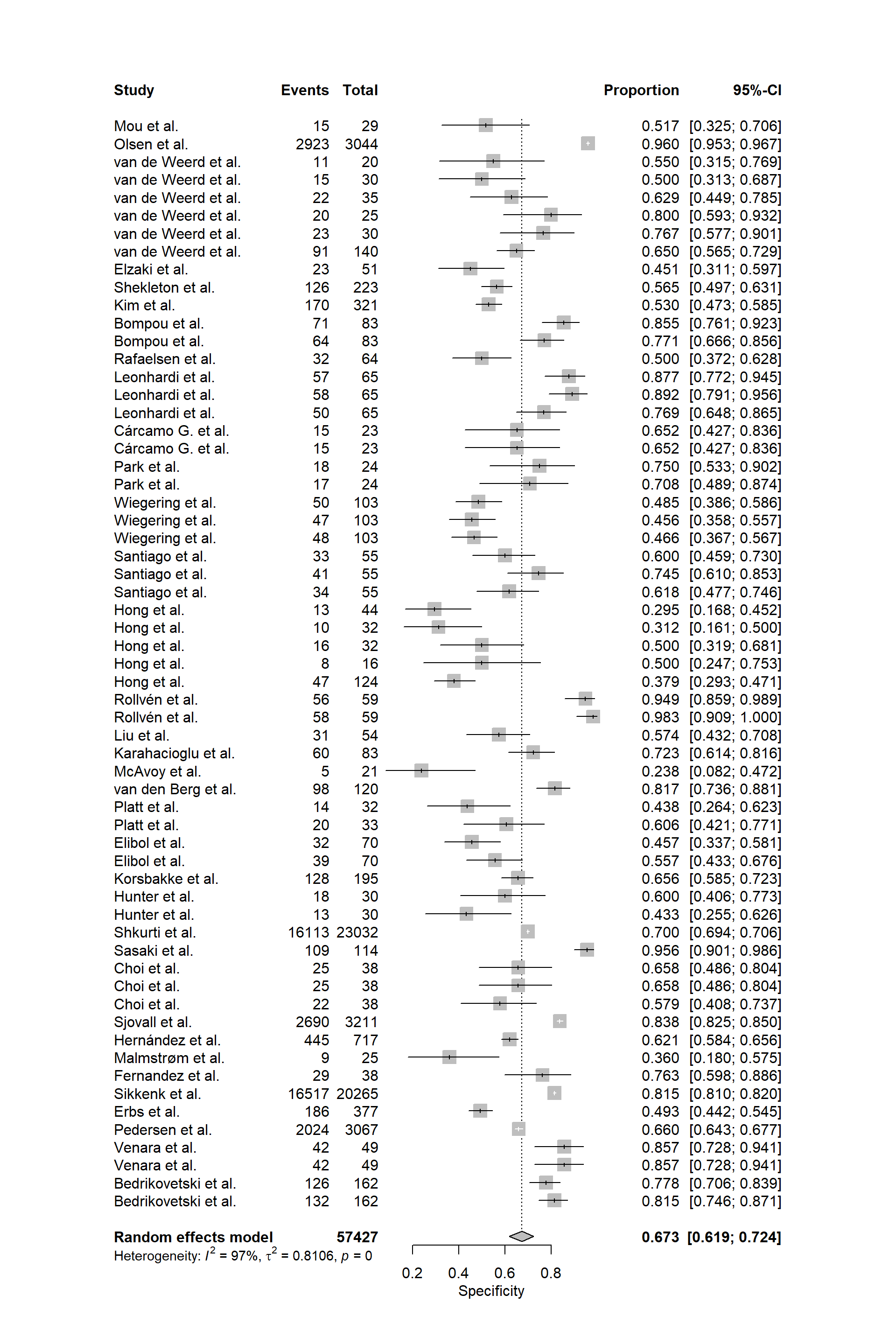


*’Events’ represent TN; ‘Total’ represents TN + FP*

## **Figure S5** Forest plot depicting sensitivity (A) and specificity (B) for differentiating EMVI+ versus EMVI-

1. Sensitivity


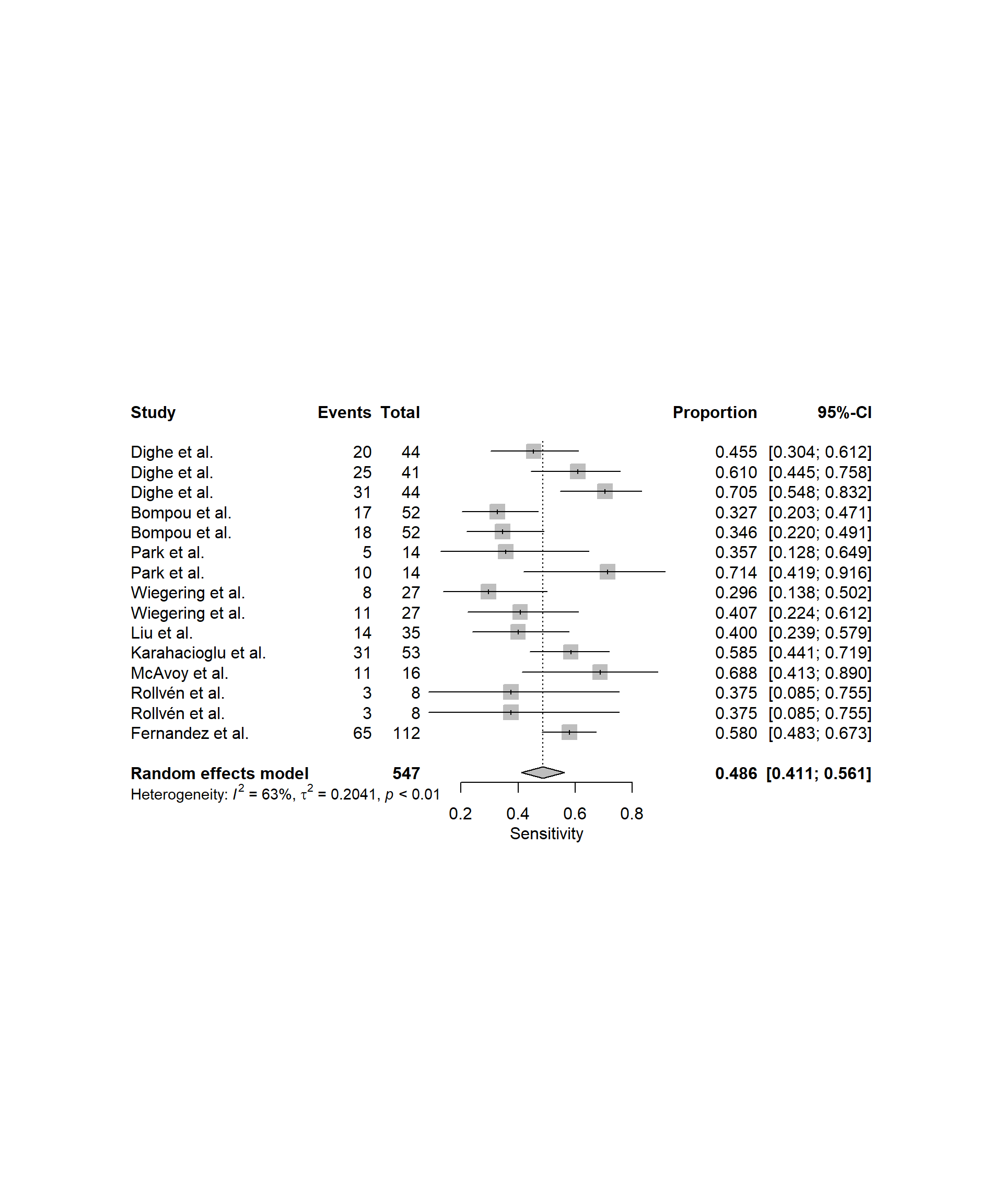


*’Events’ represent TP; ‘Total’ represents TP + FN*

1. Specificity


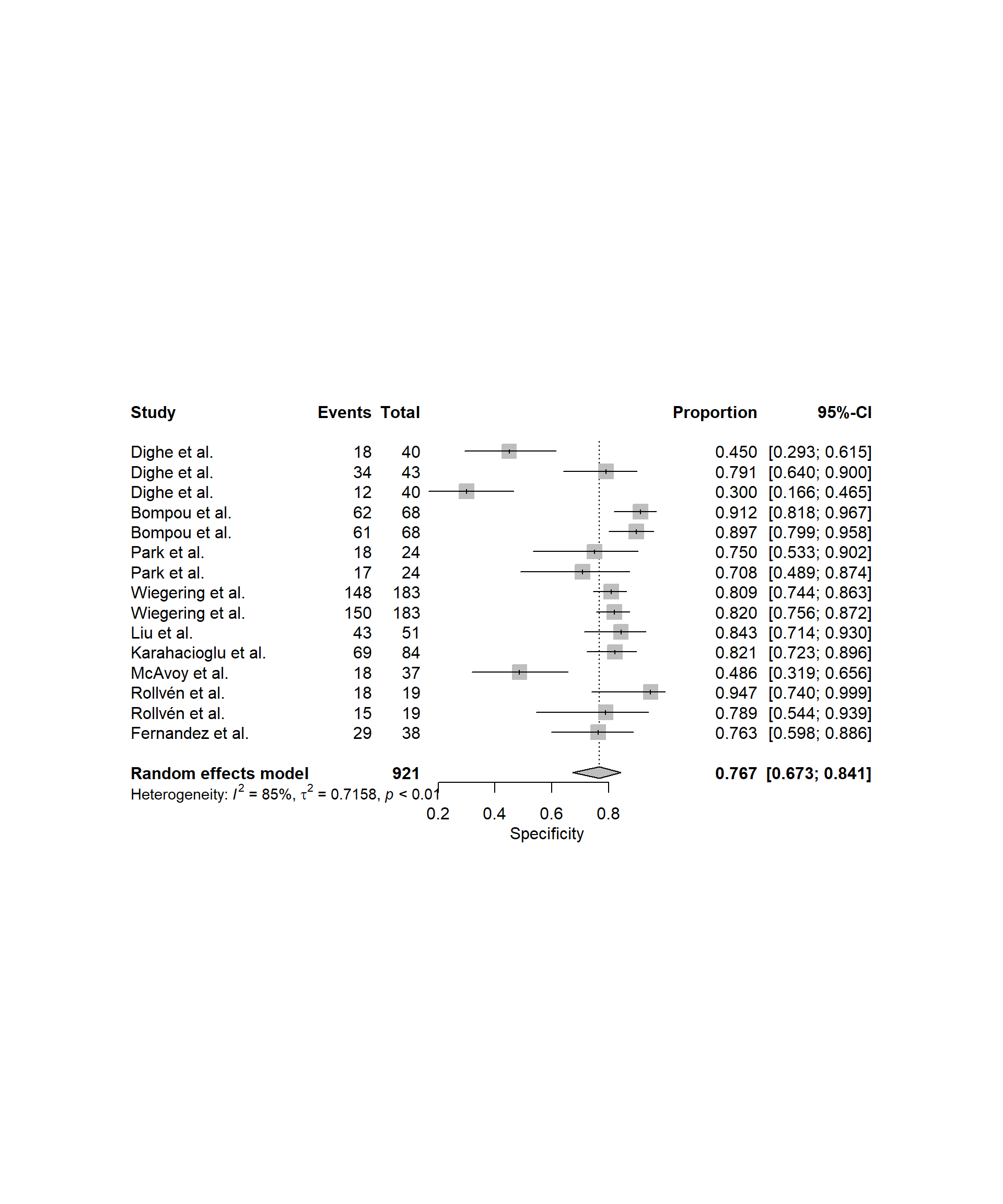


*’Events’ represent TN; ‘Total’ represents TN + FP*
